# Modelling of pricing, crashing, and coordination strategies of prefabricated construction supply Chain with power structure

**DOI:** 10.1371/journal.pone.0289630

**Published:** 2023-08-10

**Authors:** Wen Jiang, Kanfeng Shi, Lin Zhang, Weiling Jiang

**Affiliations:** College of Architecture and Urban-Rural Planning, Sichuan Agricultural University, Chengdu, Sichuan, P.R. China; Central Queensland University, AUSTRALIA

## Abstract

In the prefabricated construction industry, consumers are sensitive to the construction delivery time, and different power structures are very common. This research uses methods of Stackelberg game, Nash game and supply chain coordination, introduces a manufacturer crashing strategy into a prefabricated construction supply chain and investigates the assembler pricing, manufacturer crashing, and supply chain coordination strategies under three different power structures. It finds that adopting a crashing strategy improves the supply chain’s profit, while the dynamic wholesale price contract achieves supply chain coordination. Meanwhile, when consumer time and price sensitivity are low, it is easier to achieve high profits in the supply chain under unequal power distribution. Conversely, the supply chain profit is higher in the case of a Nash game. This study innovatively introduces the thought of power structure and crashing strategy into the prefabricated construction supply chain, and provides the optimal price and delivery time under three different power structures for prefabricated construction enterprises and realizes supply chain coordination. The conclusion can provide decision suggestions for the prefabricated construction enterprises under different competitive environments.

## 1. Introduction

The conservation of construction energy requires no delay [1]. As a sustainable construction method, prefabricated construction is an important way to save resources, reduce energy consumption and achieve green transformation for the construction industry. Compared to traditional cast-in-place construction, prefabrication can transfer some construction project stages from the field to an off-site production facility [2]. It was introduced to reduce carbon emissions, improve construction quality, and increase productivity and efficiency [3]. In recent years, the demand for prefabrication has increased for public housing and infrastructure construction [4]. However, the stability and efficiency of the prefabricated construction supply chain (PCSC), composed of manufacturers and assemblers, are prerequisites for prefabricated construction (PC). In China, prefabricated construction manufacturers (e.g., CSCEC) and assemblers (e.g., Vanke) are held by separate companies. Manufacturers produce prefabricated components (e.g., floors, stairs, and balconies) in factories and transport them to construction sites, while assemblers assemble prefabricated components on-site into PCs and sell them.

Prefabricated constructions have sustained and stable output performance in developing countries [5]. The potential instability of PCSCs can reduce or even eliminate the advantages of PCs. For example, assemblers always spend a significant amount of time waiting for components to arrive on-site. This is because manufacturers face some uncertainties and the unique challenges of PCs, such as insufficient stocks of raw materials, geometric variability, and uncertain transport routes of prefabs [6, 7]. Since material-related costs account for up to 65% of the total budget, considerable amounts of money can potentially be saved while guaranteeing the PC’s on-time delivery to maintain PCSC stability [8]. In the extant literature, many methods have been proposed to ensure that manufacturers deliver prefabricated components on time [9–11]. Such as offering incentives for early delivery and imposing penalties for late delivery. All of these studies were conducted from the perspective of assemblers. Similarly, in construction industry, the duration of a project can be shortened by methods of allocating more or efficient resources, working multiple shifts, accelerating the durations of critical activities (i.e. crashing) and extending working days, thus speeding up the delivery speed [12, 13]. In fact, owing to the efficiency of prefabricated constructions, many consumers are sensitive to delivery time. Therefore, in prefabricated construction supply chain, manufacturers can invest the crashing cost and increase their production efficiency to attract time-sensitive consumers and increase their profits. Therefore, it is of great significance to introduce the crashing strategy in the construction industry into prefabricated construction supply chain for research.

In the existing literature, few studies have investigated PCSCs considering power structure. However, the influence of power structure cannot be ignored, especially when there are large enterprises in the supply chain (which is very common in PCSCs). Large assembling enterprises, such as Balfour Beatty Construction, Gensler, and Country Garden in China, will give them more power in the supply chain because of their size. When there are strong manufacturers (e.g., EBAWE in Germany, and Changsha Broad Homes Industrial Group Co., Ltd. in China) in the PCSC, they lead the supply chain. A party with high power can take the lead in making decisions in his or her favor, thereby changing the whole supply chain’s profit [14]. Therefore, there are mainly three power structures in the current prefabricated construction market in China, which includes the manufacturer-led, the assembler-led and Nash equilibrium. By considering the three power structures in PCSCs, the results of this research are applicable to various practical situations and have better generalizability, which is of reference value to supply chain (SC) members in different competition environments.

This research studies the most widespread PCSC with power structures, consisting of an assembler and a manufacturer. The prefabricated components of a PC can be divided into standard and nonstandard components. As necessary components for all constructions, the assembler can purchase standard components in advance to reduce the uncertainty of construction time. Therefore, this study assumes that the assembler already has a stock of standard components and orders nonstandard components from the manufacturer when the order arrives. The manufacturer then produces the nonstandard components and transports them to the assembler. In a real-world scenario, the manufacturer deals with material and decides the optimal production time and the delivery time, while the assembler deals with installation and decides the price of the PC. Their decisions indirectly affect consumer demand and each other’s profits. To solve existing contradictions, this study provides a contract to coordinate the manufacturer and assembler’s decisions with three different power structures and discusses the following three questions: (1) Do manufacturers adopt a crashing strategy to produce nonstandard components? (2) What are the optimal strategies for manufacturers and assemblers in different power structures? (3) What coordination scheme can be used to resolve conflicts between assemblers and manufacturers?

To address the above problems, the paper introduces a manufacturer crashing strategy into a prefabricated construction supply chain and investigates the assembler pricing, manufacturer crashing, and supply chain coordination strategies under three different power structures. The contributions of this paper are as follows: as the existing three power structures of manufacturer-led, the assembler-led and Nash equilibrium in the prefabricated construction market in China, this paper introduces the above power structures into prefabricated construction supply chain, and constructs games models to discuss the impacts of different power structures on strategies of supply chain members. In addition, differing from the existing researches, this paper innovatively introduces the crashing strategy into prefabricated construction supply chain from the perspective of the manufacturer, and provides new management insights and decision suggestion for construction enterprises.

The remainder of this paper is organized as follows. Section 2 reviews the related literature. Section 3 describes the problem, makes assumptions, and provides notations for the problem. Section 4 presents a base model, a Nash game model and two Stackelberg game model to optimize the decision and profit of PCSC in different power structures. Section 5 illustrate a dynamic wholesale price contract to coordinate the supply chain. In Section 6, numerical analysis is used to estimate the influence of different parameters and models on supply chain profits. Section 7 summarizes conclusions of the study and explores future research directions.

## 2. Literature review

This research is related to the following three streams of literature: supply chain management (SCM) issues in PCs, the production strategies of prefabricated manufacturers, and the effect of different power structures on SCM.

### 2.1. Prefabricated construction supply chain management

Many studies investigated traditional SCM constructions. Scholars have widely recognized the importance of SCM in the construction industry, as it improves the performance of construction which usually suffered from cost and time overrun, conflicts and disputes, by coordinating independent entities [15, 16]. Furthermore, some scholars explore the SCM issues in prefabricated construction [17, 18]. However, due to the characteristics of highly fragmented construction supply chain, the application of supply chain management in the construction industry is far from satisfactory [18, 19]. Also, Kim et al. (2017) [20] pointed out that the relationship characteristics of supply chain members can significantly affect project performance in a construction supply chain. This finding may prompt construction supply chain members to improve their relationships and achieve better project performance. Nevertheless, few studies have used real-world case studies [21].

Further, only a few studies have been conducted on PCSCs as a novel construction form [22, 23]. Yang et al. (2018) [24] analyzed the characteristics of PCSCs and the current situation. They proposed a preordering strategy for the prefabricated components of assemblers. Also, Aloini et al. (2012) [18] and later Li, Shen, and Xue (2014) [25] indicated a need for future research towards the interrelationship of independent entities in the PSC from a whole supply chain perspective, as it is the key element in reducing the construction cost while satisfying its clients on time, however, have been slightly researched. In addition, some scholars also apply the PCSCM to practical engineering cases [10, 26]. Kim et al. (2016) [27] established a prefabricated supply chain time-driven cost model and applied it to a construction project. Nonetheless, compared to the extant literature on traditional construction supply chains (TCSCs), there have been few studies on PCSCs. Meanwhile, owing to the particularity of PCs (different responsibilities of supply chain members, particular forms of construction), the research conclusions on TCSCs cannot be directly applied to PCSCs. In addition, as an important member of the PCSC, little research has been conducted on the production decisions of manufacturers. Therefore, to ensure maximum profit, we study the assembler’s and manufacturer’s decisions from the supply chain perspective.

### 2.2. Producing strategy of the prefabrication manufacturer

In this study, consumer demand is related to the delivery time of the PC, which is decided by the manufacturer. The most common production strategies are make-to-order (MTO) and make-to-stock (MTS) [28], which have been explored by many studies. Rajagopalan (2002) [29], Zaerpour et al. (2008) [30] and Gad et al. (2010) [31] studied companies that needed to make product inventory and sales decisions to determine the optimal pricing, inventory, production and capacity investment strategy for the MTS program. And they proposed a strategic decision structure that could determine whether a product should choose an MTO or MTS manufacturing strategy. It provides an idea for us to study manufacturer’s producing strategy. And Bart et al. (2016) [32] compared the benefits of a hybrid planning approach that did not prioritize MTO or MTS. To optimize revenue, they found that it was important to consider whether there was a backlog of MTO orders before deciding whether to increase the MTS inventory.

All aforementioned studies were based on individual manufacturer decisions; however, in a supply chain, the manufacturer’s decision is also influenced by other supply chain members, such as assemblers. In the construction supply chain, assemblers can easily influence the manufacturers’ decisions [33]. Zhai et al. (2016) [9] introduced a lead-time hedging method for project contractors to ensure that the manufacturer delivers prefabs on time, and the manufacturer charges the contractor a crash fee [34]. Wang et al. (2012) [35] and Pan et al. (2004) [34] pointed out that the project contractor requires the manufacturer to deliver the components ahead of time short lead times helped meet customer orders in a timely manner, making the company more competitive. However, this has put considerable pressure on manufacturers, and the manufacturer charges the contractor a crash fee. This situation is also existing within companies. Hu et al. (2011) [36] studied a company in which salespeople preferred to order from the production department of their own company in advance. They derived optimal lead times and internal wholesale prices for the production department and provided management advice in different situations. This research combines the above two research approaches. The manufacturer’s decision is not only influenced by the assembler in the supply chain. They are willing to attract consumers to boost their profits by reducing their delivery times.

### 2.3. Effect of the power structure on supply chain management

In supply chains, power refers to the relative profitability of supply chain members and their ability to control the decision variables of other member operating at a different level [37]. Power structure includes equal power structure (i.e. Nash equilibrium) and unequal power structure (i.e. Manufacturer-led, Assembler-led, etc.). Many studies have examined the role of power distribution (unequal power distribution in particular) and have shown that the power structure affects market members’ decisions [38]. Unequal power structure is widely used in supply chain research. Raju and Zhang (2005) [39] studied a retailer-led Stackelberg model and investigated the coordination mechanism between retailers and suppliers. They found that a quantity discount could coordinate such supply chain structure. Equal power structures are also common in supply chains. Cai et al. (2009) [40] analyzed the influence of price and price discounts on competition among supply chain members within different power structure. They found that the vertical Nash structure is equal for supply chain members and that a balanced power structure is the most favorable for the supply chain to achieve profit maximization.

In addition, many other studies have proposed different research methods according to the power structure, which also laid the foundation for this research. Chen’s research examined the role of power relationships in sustainable supply chains and found that firms with higher channel power will gain more profits [41–43]. In the existing studies on different power structures, Stackelberg game model is commonly used to study unequal power structures, and vertical Nash model is used to analyze equal power structures [44, 45]. Zheng et al. (2019) [46] considered a closed-loop supply chain with unequal power distribution and studied optimal decisions and profits under five non-cooperative and cooperative Stackelberg game models. A variable-weighted Shapley value was proposed to coordinate the closed-loop supply chain. Shi et al. (2013) [37] examined the effect of power structure and demand models on the performance of supply chain members. They found that the effect of the power structure depends on the expected demand model and demand shock. Similarly, this research discusses three different power structures of a PCSC using different models and explores the optimal decisions for manufacturers and assemblers.

The above literature on PCSCs and the manufacturer’s production strategies forms the basis for this research. This study introduces the manufacturer crashing strategy into a PCSC and establishes profit optimization models of the PCSC under three different power structures (i.e., the Nash game model and two Stackelberg models led by the assembler and manufacturer) based on optimization theory, Nash game theory, and Stackelberg game theory. Then using the derivation, reverse solution method, and MATLAB to solve the four models (the base model and the three models). Consequently, this research formulates the pricing strategy of the assembler, the crashing strategy of the manufacturer, and the optimal profit of the prefabricated construction enterprises and supply chain under the different power structures. In the above research, the wholesale price of the prefabricated components is not determined by the manufacturer. Therefore, this research introduces a dynamic wholesale price contract and realize supply chain coordination under the three aforementioned different power structures. Finally, using the Python for numerical analysis, which not only verifies the previous conclusions, but also has some other implications. To maximize enterprises and supply chain profits, this study provides the optimal price and delivery time for PC enterprises under three different power structures and realizes supply chain coordination, which is of great significance for promoting PC development.

## 3. Methodology

### 3.1. Research process flow

**Fig 1:**
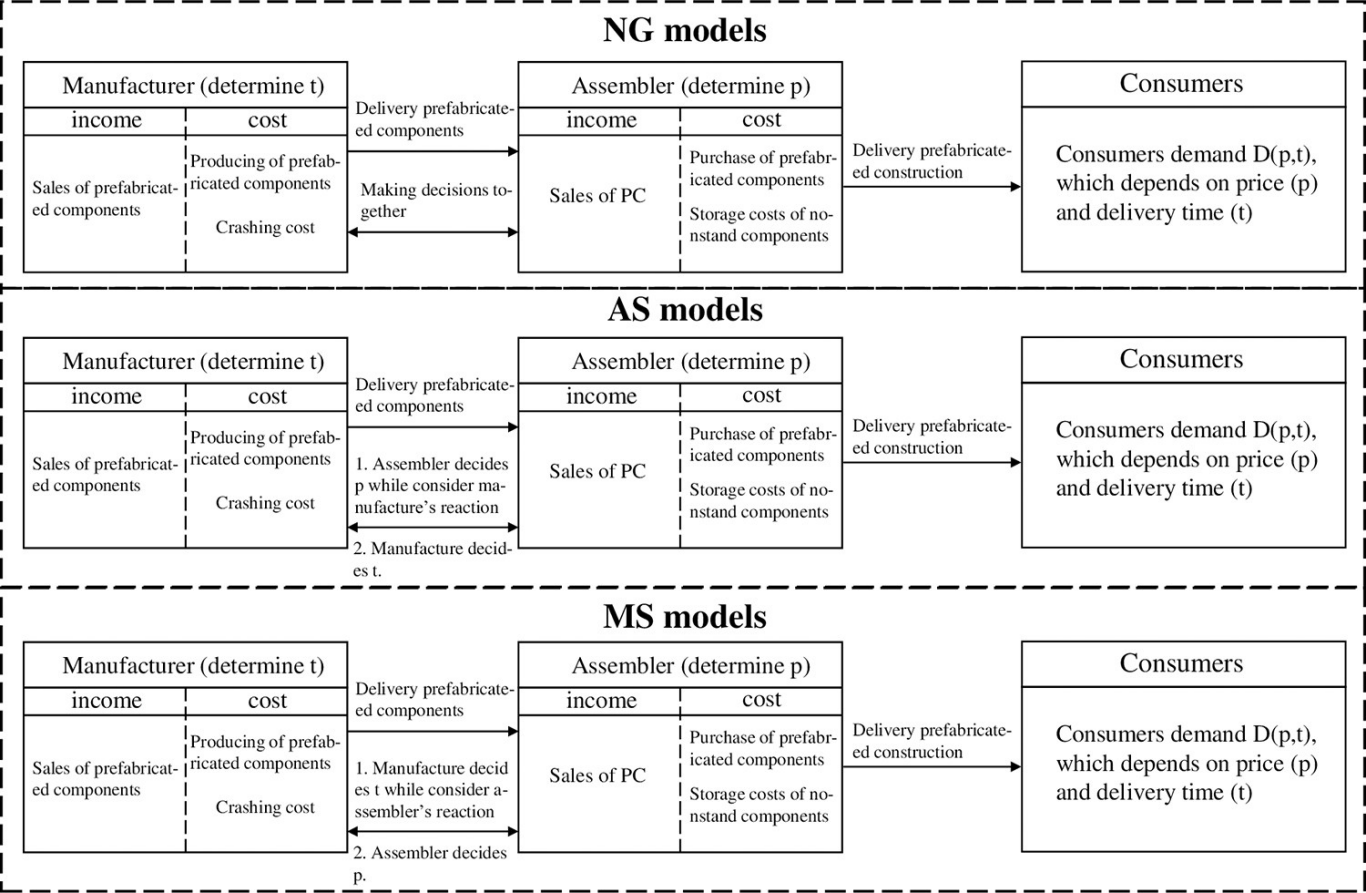
The process flows.

This study simplifies the manufacturing process of PCs to the manufacturer who produces prefabrication components off-site, and delivers them to the assembler who assembles them at the construction site. Among which, the assembler decides the price of the prefabricated construction, and the manufacturer decides the delivery time of non-standard components. In the PC industry, prefabricated components are divided into standard and nonstandard components, as reflected in this study. Based on the above operation flow of the PCSC, this paper optimizes the optimal decision of PCSC according to the specific decision order under three different power structures based on the idea of game theory. The process flow is illustrated in Fig 1.

### 3.2. Theoretical method

In PCSC. both the assembler and manufacturer face a dilemma. For the assembler, raising prices will boost profits on individual PC units, but it will also indirectly reduce the consumer demand and the manufacturers’ profits. Conversely, lower prices may reduce the assembler’s own profits. For the manufacturer, a longer production time means lower crash costs, but it reduces the consumer demand and increases the inventory cost of standard components for the assembler. Conversely, shorter production times can substantially increase crash costs. There are multiple games between them. In this research, we use the method of game theory to optimize their optimal decision and the profit.

Game theory is the study of mathematical models of strategic interactions among rational agents. It has applications in all fields of social science, as well as in operations management, systems science and computer science. Game theory considers the predicted and actual behavior of individuals in games and studies their incentive structures. The noncooperative game is used in this research. The theory of noncooperative games studies the behavior of agents in any situation where each agent’s optimal choice may depend on a forecast of the opponents’ choices. “Noncooperative” refers to choices that are based on the participant’s perceived self-interest [47].

### 3.3. Model assumptions

In this research, Stackelberg game is applied to study the PCSC with unequal power. The idea of this method is that the party with high power in the supply chain makes decisions first, and the party with less power optimizes its own profits according to the decision of the other party. Nash game model is used to study the PCSC with equal power. In this case, both parties have equal power and make decisions to maximize their own profits at the same time.

To simplify this study and make it more relevant, making the following assumptions: First, both the assembler and the manufacturer are perfectly rational in their pursuit of maximum profit. Second, the consumer demand is inversely proportional to the PC price and delivery time. Then, the assembler already has a stock of standard components and orders nonstandard components from the manufacturer when consumers’ orders arrive. The manufacturer’s crash cost is proportional to the crash time and the number of non-standard components. Finally, the consumer’s waiting time and the assembler’s standard component inventory time are equal to the production time of non-standard components.

Following Dogan and Serel (2015) [48], the consumer’s demand is dependent on price and grows negatively exponentially over time, which is $D(p,t)=ap^{-b}t^{-\lambda }(a>0,b>1\;and\;0<\lambda <1)$, where *p*^−*b*^ and *t*^−*λ*^ represent that the demand is decreasing in price and time. Considering both the crash strategy and consumer demand, what is different from previous research is that the manufacturer may voluntarily increases the production speed to maximize their profits. The initial production time of the non-standard components are *t*_1_. After adopting the crash strategies, the manufacturer’s production time for nonstandard components is *t*. When *t* = *t*_1_, there is no crash time. Simultaneously, manufacturers should bear the crash cost of *sD*(*t*_1_−*t*)(*s*>0), where *s* is the crash cost per time per nonstandard component. The holding cost that the assembler should pay is *hDt* (*h*>0). The notations of the parameters and variables used in this study are listed in Table 1.

**Table 1:** Notations of parameters and variables.

Decision variables	Descriptions
***p***	The unit price of the prefabricated construction. *ω*≤*p*
***t***	Manufacturer’s nonstandard components delivery time after crashing. 0<*t*≤*t*_1_
**Parameters**	
***D***	Consumer demand for prefabricated construction.
***ω***	Manufacturer’s wholesaling price of prefabricated components. *ω*≤*p*
***h***	The unit price of standard component stock-holding costs per unit time.
***t***_**1**_	Initial manufacturer’s nonstandard component delivery time. 0<*t*≤*t*_1_
***a***	Initial demand of the consumer.
***b***	Self-price sensitivity of consumer. *b*>1
***λ***	Self-time sensitivity of consumer. 0<*λ*<1
***s***	The unit price of nonstandard component crash costs.

## 4. Decentralized decision optimization

### 4.1. Base model

In the base model, the manufacturer does not adopt the crash strategy, and the delivery time is *t*_1_, which means that the only decision variable is the PC price, which is decided by the assembler. This research assumes that the assembler makes decisions to maximize his or her profit, regardless of the manufacturer. The pay-off function of the assembler can be described as follows:

$$\pi_{A}^{UD}(p)=(p-\omega ){ap}^{-b}{t_{1}}^{-\lambda }-ht_{1}{ap}^{-b}{t_{1}}^{-\lambda }$$ (1)

The first term is the profit from selling the construction. The second term is the holding cost of the standard components while waiting for the manufacturer to deliver the non-standard components.

There are no crash costs for the manufacturer in the base model. The manufacturer’s profit is the income from the prefabricated components they sell and can be described as follows:

$$\pi_{M}^{UD}(t_{1})=\omega {ap}^{-b}{t_{1}}^{-\lambda })$$ (2)

**Proposition 1.** The assembler’s profit function is a concave function of p, and the following equation characterizes the optimal p for the unilateral decision model:

$$p^{UD}=\frac{b(\omega +ht_{1})}{b-1}$$ (3)

The proof is in the S1 Appendix.

The base model maximizes the assembler’s profit regardless of the manufacturer. According to Proposition 1, the optimal price depends on *b*, *ω*, *h*, and *t*_1_. With an increase in the consumers’ self-price sensitivity, the optimal price decreases. A higher stock cost naturally increases the assembler’s price. When the self-price sensitivity of consumers and standard component stock-holding costs are set, the assembler finds it better to negotiate with the manufacturer about the wholesale price of components and the delivery time of non-standard components to optimize the profit.

### 4.2. Crashing models with power structures

This subsection discusses the models under three different power structures of the PCSC: the manufacturer Stackelberg (MS) model, the Nash game (NG) model, and the assembler Stackelberg (AS) model.

#### 4.2.1. The manufacturer Stackelberg (MS) model

In this scenario, the manufacturer has more power than the assembler, which is sufficient to determine the crash time of nonstandard components in the first stage. The delivery time of nonstandard components is *t*. Subsequently, the assembler determines the optimal price to maximize his or her profit with the given crash time.

In addition to all the conditions and settings described in the base model, the assembler decides the price of PC *p* for the manufacturer. Simultaneously, the manufacturer chooses the crash time of the nonstandard component production *t* to the assembler. The objective functions for the assembler and manufacturer are:

$$\pi_{A}^{MS}(p)=(p-\omega )ap^{-b}t^{-\lambda }-htap^{-b}t^{-\lambda }$$ (4)

$$\pi_{M}^{MS}(t)=\omega ap^{-b}t^{-\lambda }-s(t_{1}-t)ap^{-b}t^{-\lambda }$$ (5)

**Proposition 2**. *Under the manufacturer Stackelberg model*, *the assembler’s optimal price of the PC is*:

$$p^{MS}=\frac{b}{b-1}(\omega +\frac{\omega +bh(\omega -st_{1})}{b-1})$$ (6)

and the delivery time for the manufacturer is:

$$t^{MS}=\frac{\omega +bh(\omega -st_{1})}{h(b-1)}$$ (7)

The proof is in the S1 Appendix.

The delivery time increases with an increase in the PC price. A high price of components makes the manufacturer earn more profit from a single PC, and he or she is willing to increase the consumer’s demand by shortening the delivery time to increase his or her profit. For the assembler, with an increase in delivery time, the optimal price of the PC increases, and the ratio is $\frac{b}{(b-1)h}$. It is easy to see that when the manufacturer leads the Stackelberg game, the assembler has to raise prices even more to maintain his or her profit with high self-price sensitivity of consumers (*b*) and stock costs (*h*).

#### 4.2.2. The Nash game (NG) model

In this scenario, the assembler and manufacturer have equal power. Since consumer demand grows exponentially over time, the manufacturer can improve his or her profit and improve the assembler’s profit unintentionally by saving the delivery time of nonstandard components. The assembler simultaneously decides the optimal price to maximize his or her profit simultaneously. A Nash game is played for this fair power setting, and the profit functions for each player are the same as those in (4) and (5) in the MS model.

It can show that the Nash equilibrium exists, and that optimal strategies can be obtained by computing the Nash equilibrium described in the following proposition:

**Proposition 3.** Under the Nash game setting for the manufacturer and assembler, the optimal strategies for both parties are determined by the Nash equilibrium, characterized by the following equations:

$$p^{NG}=\frac{b}{b-1}(\omega +\frac{h\lambda (\omega -st_{1})}{s(1-\lambda )})$$ (8)

$$t^{NG}=\frac{\lambda (\omega -st_{1})}{s-\lambda s}$$ (9)

The proof is in the S1 Appendix.

As observed in Proposition 3, the optimal price of the PC decreases with the increase in self-time-sensitivity. This is the same as in the base model, and it can explain that the assembler deals with consumers directly in both models. For the manufacturer, the reason why the optimal delivery time of nonstandard components increases with the price of the components is the same as that in Proposition 2. Thus, it not repeats this process here.

#### 4.2.3. The assembler Stackelberg (AS) model

In this scenario, the assembler has more power than the manufacturer, and he or she is powerful enough to decide the price in the first stage. Subsequently, the manufacturer determines the optimal delivery time to maximize his or her profit at the given PC price. The profit functions for each player are the same as those in Eqs (4) and (5) in the MS model.

**Proposition 4.** Under the AS model, the optimal delivery time of the nonstandard components is:

$$t^{AS}=\frac{\lambda (st_{1}-\omega )}{\lambda s-s}$$ (10)

The optimal price of PC for assemblers is:

$$p^{AS}=\frac{b}{b-1}(\omega +\frac{h\lambda (st_{1}-\omega )}{s(\lambda -1)})$$ (11)

The proof is in the S1 Appendix.

The PC price increases with stock-holding costs and decreases with the wholesale price of nonstandard components. Hence, the assembler would be better off reducing stock-holding costs by improving the inventory strategy and negotiating with the manufacturer about the price of non-standard components before making decisions.

The AS model supply chain decision is the same as that in the NG model. This research analyzes and interprets the results as follows. In the NG and AS models, the assembler deals with consumers directly and considers only consumer demand when making decisions, and the optimal retail price is the same. The delivery time is the same because the manufacturer can only determine the delivery time according to the same given PC price. Only in the MG model, the manufacturer decides the delivery time in the first stage while dealing directly with consumers, and different supply chain decisions are made. Although the assembler deals with consumers directly in both the NG and AS models, the supply chain’s decision order is completely different. In the NG model, the powers of the assembler and manufacturer are equal, and they make decisions at the same time. However, in the AS model, the assembler has more power and makes decisions in the first stage. Therefore, the competitive environments applicable to the two models are completely different.

### 4.3. The comparison

This scenario compares the performances of the three crash models with the base model and with each other. The features and performance of the three crash models and the base model are presented in Table 2.

**Table 2:** Features and performance of three models.

Feature/Performance	UD model	MS model	NG model	AS model
Power structure	Assembler has full power	Manufacturer is powerful	Equal power	Assembler is powerful
Decision-making order	Assembler decides first	Manufacturer decides first	At the same time	Assembler decides first
The party faces consumer	Assembler	Manufacturer	Assembler	Assembler
Optimal price	$\omega {ap}^{-b}{t_{1}}^{-\lambda }$	$\frac{b}{b-1}(\omega +\frac{\omega +bh(\omega -st_{1})}{b-1})$	$\frac{b}{b-1}(\omega +\frac{h\lambda (\omega -st_{1})}{s(1-\lambda )})$	$\frac{b}{b-1}(\omega +\frac{h\lambda (st_{1}-\omega )}{s(\lambda -1)})$
Optimal time	$\frac{b(\omega +ht_{1})}{b-1}$	$\frac{\omega +bh(\omega -st_{1})}{h(b-1)}$	$\frac{\lambda (\omega -st_{1})}{s-\lambda s}$	$\frac{\lambda (st_{1}-\omega )}{\lambda s-s}$

#### 4.3.1. The comparison with base model

In this scenario, some results are observed by comparing the unit price, delivery time, and each party’s profit between the three different crash models and the basic model.

**Proposition 5.** By comparing the MS model with the unilateral decision model, this research has the following results:

1. When $t_{1}\geq \frac{\omega (bh+1)}{h(bs+b-1)}$, *p*^*MS*^≤*p*^*UD*^; when $t_{1}<\frac{\omega (bh+1)}{h(bs+b-1)}$, *p*^*MS*^>*p*^*UD*^.
2. When $t_{1}\geq \frac{\omega (bh+1)}{h(bs+b-1)}$, $\pi_{A}^{MS}\geq \pi_{A}^{UD}$, $\pi_{M}^{MS}\geq \pi_{M}^{UD}$ and *π*^*MS*^≥*π*^*UD*^; when $t_{1}<\frac{\omega (bh+1)}{h(bs+b-1)}$, $\pi_{A}^{MS}<\pi_{A}^{UD}$, $\pi_{M}^{MS}<\pi_{M}^{UD}$ and *π*^*MS*^<*π*^*U*^.

The proof is in the S1 Appendix.

From Proposition 5, it’s found that under the MS model, the manufacturer has direct contact with consumers. When *t*_1_ is high, there is a wide time frame for the manufacturer to shorten the production time of the nonstandard components. At this point, consumer demand increases, and the assembler’s profit is stable. Thus, the assembler is willing to reduce prices to attract more consumers, and the profits of all parties naturally increase. Conversely, when *t*_1_ is low, the manufacturer has no room to shorten the delivery time, and the assembler must increase the price to obtain his or her profit. When $t_{1}<\frac{\omega (bh+1)}{h(bs+b-1)}$, the delivery time is greater than *t*_1_, which is not accepted by consumers. Consequently, the profit of each member decreases.

**Proposition 6.** By comparing the NG model with the unilateral decision model, it obtains the following results:

1. When $t_{1}\geq \frac{\lambda \omega }{s}$, *p*^*NG*^≤*p*^*UD*^; when $t_{1}<\frac{\lambda \omega }{s}$, *p*^*NG*^>*p*^*UD*^.
2. When $t_{1}\geq \frac{\lambda \omega }{s}$, $\pi_{A}^{NG}\geq \pi_{A}^{UD}$, $\pi_{M}^{NG}\geq \pi_{M}^{UD}$ and *π*^*NG*^≥*π*^*UD*^; when $t_{1}<\frac{\lambda \omega }{s}$, $\pi_{A}^{NG}<\pi_{A}^{UD}$, $\pi_{M}^{NG}<\pi_{M}^{UD}$ and *π*^*NG*^<*π*^*UD*^.

The proof is in the S1 Appendix.

It finds that when the supply chain has equal power structure, the assembler deals directly with consumers. When the price of the components is low, the assembler has a high profit from a single PC, and an increase in unit price brings less benefit than an increase in unit output. Here, the assembler is willing to reduce PC prices to increase the consumer demand. When *t*_1_ is high, the manufacturer has sufficient capacity and is willing to shorten the delivery time and increase each member’s profit. When $t_{1}<\frac{\lambda \omega }{s}$, the delivery time is greater than *t*_1_, which is not acceptable by consumers, and the profit of each member decreases.

**Proposition 7.** By comparing the AS model with the unilateral decision model, it obtains the following results:

1. When $t_{1}\geq \frac{\lambda \omega }{s}$, *p*^*NG*^≤*p*^*UD*^; when $t_{1}<\frac{\lambda \omega }{s}$, *p*^*NG*^>*p*^*UD*^.
2. When $t_{1}\geq \frac{\lambda \omega }{s}$, $\pi_{A}^{AS}\geq \pi_{A}^{UD}$, $\pi_{M}^{AS}\geq \pi_{M}^{UD}$ and *π*^*AS*^≥*π*^*UD*^; when $t_{1}<\frac{\lambda \omega }{s}$, $\pi_{A}^{AS}<\pi_{A}^{UD}$, $\pi_{M}^{AS}<\pi_{M}^{UD}$ and *π*^*AS*^<*π*^*UD*^.

The proof is in the S1 Appendix.

From Proposition 7, this research concludes that once the power of the assembler is greater than or equal to that of the manufacturer (the NG or AS models), they have a consistent optimal decision. The assembler Stackelberg game optimizes the supply chain profits by reducing the price and delivery time of PCs. When $t_{1}<\frac{\lambda \omega }{s}$, the delivery time is greater than *t*_1_, which is not acceptable by consumers, and consequently the profit of each member decreases.

#### 4.3.2 The comparison between three power structures

In this scenario, three results are observed by comparing the unit price, delivery time, and each party’s profit in the NG model and the two Stackelberg game models.

**Proposition 8**. Comparing the Nash game model and the two Stackelberg models, it finds that:

1. When $\frac{\lambda h(st_{1}-\omega )(b-1)}{\omega s(\lambda -1)+bhs(\lambda -1)(\omega -st_{1})}\geq 1$, *t*^*NG*^ = *t*^*AS*^≥*t*^*MS*^ and when $\frac{\lambda h(st_{1}-\omega )(b-1)}{\omega s(\lambda -1)+bhs(\lambda -1)(\omega -st_{1})}\leq 1$, *t*^*NG*^ = *t*^*AS*^≤*t*^*M*^;
2. When $\frac{\lambda h(st_{1}-\omega )(b-1)}{\omega s(\lambda -1)+bhs(\lambda -1)(\omega -st_{1})}\geq 1$, *p*^*NG*^ = *p*^*AS*^≥*p*^*MS*^ and when $\frac{\lambda h(st_{1}-\omega )(b-1)}{\omega s(\lambda -1)+bhs(\lambda -1)(\omega -st_{1})}\leq 1$, *p*^*NG*^ = *p*^*AS*^≤*p*^*MS*^;
3. When $\frac{\lambda h(st_{1}-\omega )(b-1)}{\omega s(\lambda -1)+bhs(\lambda -1)(\omega -st_{1})}\geq 1$, $\pi_{A}^{NG}=\pi_{A}^{AS}\leq \pi_{A}^{MS}$, $\pi_{M}^{NG}=\pi_{M}^{AS}\leq \pi_{M}^{MS}$ and *π*^*NG*^ = *π*^*AS*^≤*π*^*UD*^; when $\frac{\lambda h(st_{1}-\omega )(b-1)}{\omega s(\lambda -1)+bhs(\lambda -1)(\omega -st_{1})}\leq 1$, $\pi_{A}^{NG}=\pi_{A}^{AS}\geq \pi_{A}^{MS}$, $\pi_{M}^{NG}=\pi_{M}^{AS}\geq \pi_{M}^{MS}$ and *π*^*NG*^ = *π*^*AS*^≥*π*^*UD*^.

The proof is in the S1 Appendix.

Both of the Nash game and Stackelberg game models can optimize the supply chain’s profit with different power structures. However, different model performances depend on objective conditions, such as consumer self-sensitivity, holding costs, and the wholesale price of components. High prices and long delivery time of PCs reduce consumer demand and affect the supply chain’s profit. Thus, regardless of who is the dominant party, it will choose the proper price and delivery time of the PC to increase the supply chain profits unintentionally.

## 5. Coordination with dynamic wholesale price contract

### 5.1. Global optimal model

In the global optimal model, the entire supply chain is regarded as a whole system and optimal decisions are made to maximize public welfare. The total profit can be described as:

$$\pi^{GO}(p,t)=pap^{-b}t^{-\lambda }-htap^{-b}t^{-\lambda }-s(t_{1}-t)ap^{-b}t^{-\lambda }$$ (12)

The first term is the profit from selling the construction. The second term is the stock-holding cost of standard components, while the assembler waits for the manufacturer to deliver non-standard components. The third term is the crash cost while producing non-standard components.

**Proposition 9.** Under the global optimal setting for a manufacturer and assembler, the optimal strategies for both parties are characterized by the following equations:

$$p^{GO}=\frac{bst_{1}}{b+\lambda -1}$$ (13)

$$t^{GO}=\frac{\lambda st_{1}}{\left(b+\lambda -1)(s-h\right)}$$ (14)

Moreover, the optimal profit of the supply chain is:

$$\pi^{GO}=\frac{a{\left(b+\lambda -1\right)}^{b+\lambda -1}{\left(s-h\right)}^{\lambda }}{b^{b}\lambda^{\lambda }s^{b+\lambda -1}{t_{1}}^{b+\lambda -1}}$$

The proof is in the S1 Appendix.

The optimal price and delivery time of the PC in the global optimal model depend on *b*, *h*, *λ* and *t*_1_. Assemblers and manufacturers pursue PC profit maximization regardless of their own profit. Although the profits of the assembler and manufacturer may decrease due to their decisions, they sacrifice their profits to optimize the supply chain profits.

**Proposition 10.** The profit of the entire SC in different game models is in line with the following conclusions:

$$\pi^{GO}>\pi^{NG},\pi^{GO}>\pi^{AS}and\pi^{GO}>\pi^{MS}$$

The proof is in the S1 Appendix.

Proposition 10 shows that the PCSC’s total profit with centralized decision-making (GO model) is higher than that of decentralized decision-making. This is because, under decentralized decision-making, each member’s goal is to pursue their own profit. Meanwhile, the abuse of power by the assembler and manufacturer may bring huge profits to the supply chain in the short term. However, this is not conducive to expanding the product sales market, and will ultimately reduce the total profit of the supply chain in the long term. From the above analysis, under decentralized decision-making, the price and delivery time of PCs are not in an optimal state. Specifically, under decentralized decision-making, there is no optimal price and delivery time in the supply chain to promote further cooperation between supply chain members to achieve profits in the global optimal model. In the next subsection, this research constructs the cooperation and coordination model to achieve the supply chain’s optimal profit without losing each member’s profit with centralized decision-making.

### 5.2. Coordination strategies

Centralized decision-making requires PCSCM to make a uniform decision. However, since manufacturers and assemblers are independent economic entities, they will not work together voluntarily without being motivated. Thus, a coordination model is constructed to encourage participation by each member, with their goals being the maximization of the total profit of the supply chain and ensure that each member’s profit is not less than that of the decentralized decision-making model.

This subsection introduces a dynamic wholesale price contract to coordinate the manufacturer and assembler’s decisions. Compared with other coordination contracts, the dynamic wholesale price contract is more flexible in coordinating a decentralized decision-making supply chain with an unequal power structure [49] (Fang. 2018). It can create a win-win transaction for both the manufacturer and assembler [50]. Arnab et al. (2020) [51] designed a dynamic wholesale price contract, and coordinated a green apparel supply chain with unequal power structure. The research methods and results have reference values for this research.

In this coordination model, the manufacturer and assembler make commitment that the assembler’s wholesale price of components is related to the PC’s sales volume. It assumes that the wholesale price is a function of consumer demand, and it can be expressed as $\omega (p,t)=c_{0}+\frac{\theta }{D(p,t)}$, where *c*_0_ is the cost of prefabricated components for the manufacturer and *θ* is a coordinate parameter. Manufacturers can customize dynamic wholesale prices to incentivize assemblers to adjust PC prices and increase consumer demand. First, this research discusses the MS game, and substitutes $\omega (p,t)=c_{0}+\frac{\theta }{D(p,t)}$ into Formulas (4) and (5). The profit functions for each player under dynamic wholesale price contracts are as follows:

$$\pi_{A}^{CMS}(p)=(p-c_{0}-ht)ap^{-b}t^{-\lambda }-\theta$$ (15)

$$\pi_{M}^{CMS}(t)=(c_{0}-s(t_{1}-t))ap^{-b}t^{-\lambda }+\theta$$ (16)

It can then calculate the optimal price and delivery time, and the proof is basically the same as that of Proposition 2. Thus, it does not repeat it here.


$$p^{CMS}=\frac{b}{b-1}(c_{0}+\frac{c_{0}+bh(c_{0}-st_{1})}{b-1})$$

$$t^{CMS}=\frac{c_{0}+bh(c_{0}-st_{1})}{h(b-1)}$$

Then substituting *p*^*CMS*^ and *t*^*CMS*^ into Formulas (16) and (17), and we have $\pi_{A}^{CMS}(\theta )$ and $\pi_{M}^{CMS}(\theta )$.


$$\pi_{A}^{CMS}(\theta )=\frac{ah^{\lambda }{\left(b-1\right)}^{\lambda +2b-2}}{b^{b}{\left(bc_{0}+bh(c_{0}-st_{1})\right)}^{b-1}{\left(c_{0}+bh(c_{0}-st_{1})\right)}^{\lambda }}-\theta$$

$$\pi_{M}^{CMS}(\theta )=\frac{ah^{\lambda -1}{\left(b-1\right)}^{\lambda +2b-1}(c_{0}h(b-1)-sht_{1}(b-1+bs)+sc_{0}(bh+1))}{b^{b}{\left(bc_{0}+bh(c_{0}-st_{1})\right)}^{b}{\left(c_{0}+bh(c_{0}-st_{1})\right)}^{\lambda }}+\theta$$

The total profit of the supply chain $\pi^{CMS}=\pi_{A}^{CMS}(\theta )+\pi_{M}^{CMS}(\theta )$.


$$\pi^{CMS}=\frac{ah^{\lambda }{\left(b-1\right)}^{\lambda +2b-2}(bc_{0}+c_{0}h+sht_{1}(bs-1)-sc_{0}(bh+1))}{b^{b}{\left(bc_{0}+bh(c_{0}-st_{1})\right)}^{b}{\left(c_{0}+bh(c_{0}-st_{1})\right)}^{\lambda }}$$

**Proposition 11.** *When θ*∈[*θ*_*min*_, *θ*_*max*_], *the profit of the supply chain under the manufacturer Stackelberg game with a dynamic wholesale price contract realizes the profit under the global optimal model*. *Then*, *the dynamic wholesale price contract coordinates the supply chain under the manufacturer’s Stackelberg game*.


$$\theta_{min}=\frac{ah^{\lambda -1}{\left(b-1\right)}^{\lambda +2b-1}(\omega h(b-1)-sht_{1}(b-1+bs)+s\omega (bh+1))}{b^{b}{\left(b\omega +bh(\omega -st_{1})\right)}^{b}{\left(\omega +bh(\omega -st_{1})\right)}^{\lambda }}-\frac{a(c_{0}(b+\lambda -1)(s-h)-s(b-1)(s-h)t_{1}+h\lambda st_{1}){\left(b+\lambda -1\right)}^{b+\lambda -1}{\left(s-h\right)}^{\lambda -1}}{b^{b}\lambda^{\lambda }s^{b+\lambda }{t_{1}}^{b+\lambda }}$$

$$\theta_{max}=\frac{a(bst_{1}(s-h)-c_{0}(b+\lambda -1)(s-h)-h\lambda st_{1}){\left(b+\lambda -1\right)}^{b+\lambda -1}{\left(s-h\right)}^{\lambda -1}b^{b}{\left(b\omega +bh(\omega -st_{1})\right)}^{b-1}{\left(c_{0}+bh(\omega -st_{1})\right)}^{\lambda }}{b^{2b}\lambda^{\lambda }s^{b+\lambda }{t_{1}}^{b+\lambda }{\left(b\omega +bh(\omega -st_{1})\right)}^{b-1}{\left(c_{0}+bh(\omega -st_{1})\right)}^{\lambda }}$$

$$—ah^{\lambda }{\left(b-1\right)}^{\lambda +2b-2}b^{b}\lambda^{\lambda }s^{b+\lambda }{t_{1}}^{b+\lambda }b^{2b}\lambda^{\lambda }s^{b+\lambda }{t_{1}}^{b+\lambda }{\left(b\omega +bh(\omega -st_{1})\right)}^{b-1}{\left(c_{0}+bh(\omega -st_{1})\right)}^{\lambda }$$

The proof is in the S1 Appendix.

Proposition 11 provides the *θ* interval that can be accepted by each party, as none of them are worse off within this interval. Simultaneously, the PCSC’s profit is as good as that of the global optimal model. In other words, the supply chain in the MS model is coordinated. However, the actual value of *θ* also relies on negotiation between the manufacturer and assembler. For example, a smaller *θ* value represents a lower profit for the manufacturer. If the manufacturer’s negotiating ability is strong, a larger *θ* can be achieved, and vice versa.

To coordinate the assembler Stackelberg game, it assumes that the wholesale price is a negative function of consumer demand, which can be expressed as $\omega (p,t)=c_{0}+\frac{l}{D(p,t)}$, where *c*_0_ is the cost of prefabricated components for the manufacturer (*c*_0_<*ω*). Similar to the manufacturer’s Stackelberg game coordination, the profit functions for each player under dynamic wholesale price contracts are as follows:

$$\pi_{A}^{CMS}(p,t)=(p-c_{0}-ht)ap^{-b}t^{-\lambda }-l$$ (17)

$$\pi_{M}^{CMS}(p,t)=(c_{0}-s(t_{1}-t))ap^{-b}t^{-\lambda }+l$$ (18)

It can then calculate the optimal price and delivery time, and the proof is basically the same as that of Proposition 2. Thus, it does not repeat it here.


$$p^{CAS}=\frac{b}{b-1}(c_{0}+\frac{h\lambda (st_{1}-c_{0})}{s(\lambda -1)})$$

$$t^{CAS}=\frac{\lambda (st_{1}-c_{0})}{\lambda s-s}$$

Then substituting *p*^*CAS*^ and *t*^*CAS*^ into Formulas (18) and (19), and have $\pi_{A}^{CAS}(l)$ and $\pi_{M}^{CAS}(l)$.


$$\pi_{A}^{CAS}(l)=\frac{as^{\lambda +b-1}{\left(b-1\right)}^{b-1}{\left(1-\lambda \right)}^{\lambda +b-1}}{b^{b}\lambda^{\lambda }{\left(c_{0}s(1-\lambda )+\lambda h(st_{1}-c_{0})\right)}^{b-1}{\left(st_{1}-c_{0}\right)}^{\lambda }}-l$$

$$\pi_{M}^{CAS}(l)=\frac{a{\left(b-1\right)}^{b}s^{\lambda +b}{\left(1-\lambda \right)}^{\lambda +b-1}((1-2\lambda )c_{0}+st_{1}(2\lambda -1))}{b^{b}\lambda^{\lambda }{\left(c_{0}s(1-\lambda )+\lambda h(st_{1}-c_{0})\right)}^{b}{\left(st_{1}-c_{0}\right)}^{\lambda }}+l$$

The total profit of the supply chain $\pi^{CAS}=\pi_{A}^{CAS}(l)+\pi_{M}^{CAS}(l)$.


$$\pi^{CAS}=\frac{as^{\lambda +b-1}{\left(1-\lambda \right)}^{\lambda +b-1}{\left(b-1\right)}^{b-1}(c_{0}s(1-\lambda )+(s(b-1)(2\lambda -1)+\lambda h)(st_{1}-c_{0}))}{b^{b}\lambda^{\lambda }{\left(c_{0}s(1-\lambda )+\lambda h(st_{1}-c_{0})\right)}^{b}{\left(st_{1}-c_{0}\right)}^{\lambda }}$$

**Proposition 12.** *When l*∈[*l*_*min*_, *l*_*max*_], the SC profit under the AS model with a dynamic wholesale price contract realizes the profit under the global optimal model. Then, the dynamic wholesale price contract coordinates the supply chain under the assembler Stackelberg game.

$$l_{min}=\frac{a{\left(b-1\right)}^{b}s^{\lambda +b}{\left(1-\lambda \right)}^{\lambda +b-1}(\left(1-2\lambda )\omega +st_{1}(2\lambda -1\right))}{b^{b}\lambda^{\lambda }{\left(\omega s(1-\lambda )+\lambda h(st_{1}-\omega )\right)}^{b}{\left(st_{1}-\omega \right)}^{\lambda }}-\frac{a(c_{0}(b+\lambda -1)(s-h)-s(b-1)(s-h)t_{1}+h\lambda st_{1}){\left(b+\lambda -1\right)}^{b+\lambda -1}{\left(s-h\right)}^{\lambda -1}}{b^{b}\lambda^{\lambda }s^{b+\lambda }{t_{1}}^{b+\lambda }}$$

$$l_{max}=\frac{a(bst_{1}(s-h)-c_{0}(b+\lambda -1)(s-h)-h\lambda st_{1}){\left(b+\lambda -1\right)}^{b+\lambda -1}{\left(s-h\right)}^{\lambda -1}}{b^{b}\lambda^{\lambda }s^{b+\lambda }{t_{1}}^{b+\lambda }}-\frac{as^{\lambda +b-1}{\left(b-1\right)}^{b-1}{\left(1-\lambda \right)}^{\lambda +b-1}}{b^{b}\lambda^{\lambda }{\left(\omega s(1-\lambda )+\lambda h(st_{1}-\omega )\right)}^{b-1}{\left(st_{1}-\omega \right)}^{\lambda }}$$

The proof is in the S1 Appendix.

Similar to Proposition 11, Proposition 12 provides the available interval of *l*. The PCSC under the AS and NG models is coordinated. Thus far, it has found that dynamic wholesale price contracts can coordinate PCSCs under the three different power structures. Further, manufacturers and assemblers only need to discuss coordination parameters *θ* or *l* that meet the above reasonable range to achieve supply chain profits under centralized decisions.

## 6. Numerical studies

This section compares the performances of the base model, the two Stackelberg models, and the global optimal model. It does not discuss the Nash game model here because it has the same result as the assembler Stackelberg model. The aim is to determine the model that should be adopted to satisfy the needs of supply chain members and consumers under different power structures. Numerical analysis is also provided to verify the previous conclusions and provide more insights. As long as the parameter settings conform to all the assumptions in this paper, the conclusion of numerical analysis is valid, and the overall conclusion of the paper can be proved.

In this Section, numerical analysis is performed based on a sensitivity analysis of two parameters of these models: consumer self-price sensitivity (*b*) and consumer self-time sensitivity (*λ*). It compares the unit price, delivery time, and profit of each part in different models. Referring to correlative studies [9, 43] and meeting all the assumptions above, it initially sets *ω* = 13, *h* = 0.8, *t*_1_ = 10, *a* = 30000, *b* = 1.5, *λ* = 0.67, and *s* = 2.1 in which *ω* refers to Zhai et al.(2016) [9], *h* and *s* refer to Chen et al.(2017) [43], while the remaining parameters are set according to our models. To study the impact of *λ* and *b* on delivery time, price, and profit, we selected 10 values of *λ* and *b*, among which *λ*∈[0.655, 0.7] and *b*∈[1.46, 1.55]. The support data for this section were saved in the first set of data of S1 File.

### 6.1. The impact of consumer sensitivity on supply chain members

**Fig 2:**
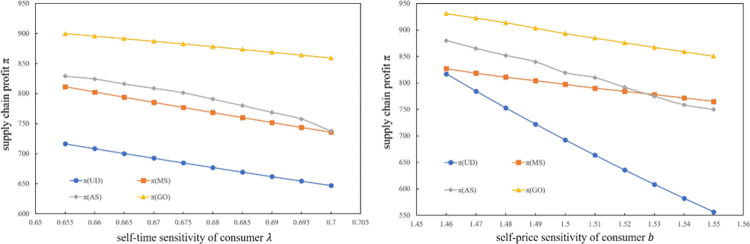
The effect of *λ* and *b* on *π*.

This subsection investigates the effect of parameters *b* and *λ* on the total profit of the supply chain *π*, the profit of assembler *π*_*A*_, and the profit of the manufacturer *π*_*M*_. Fig 2 shows that the supply chain profits under the three cooperative models are higher than those of the assembler individual decision model. Compared to the three game models, the global optimal model significantly increases the supply chain’s profit. From the given initial data, the AS and NG models found to be more efficient than the MS model most of the time. However, the PCSC’s profit in the AS model decreases faster than that in the MS model, with the increase in *b*. This is reasonable because the profit of assemblers is higher than that of manufacturers.

**Fig 3:**
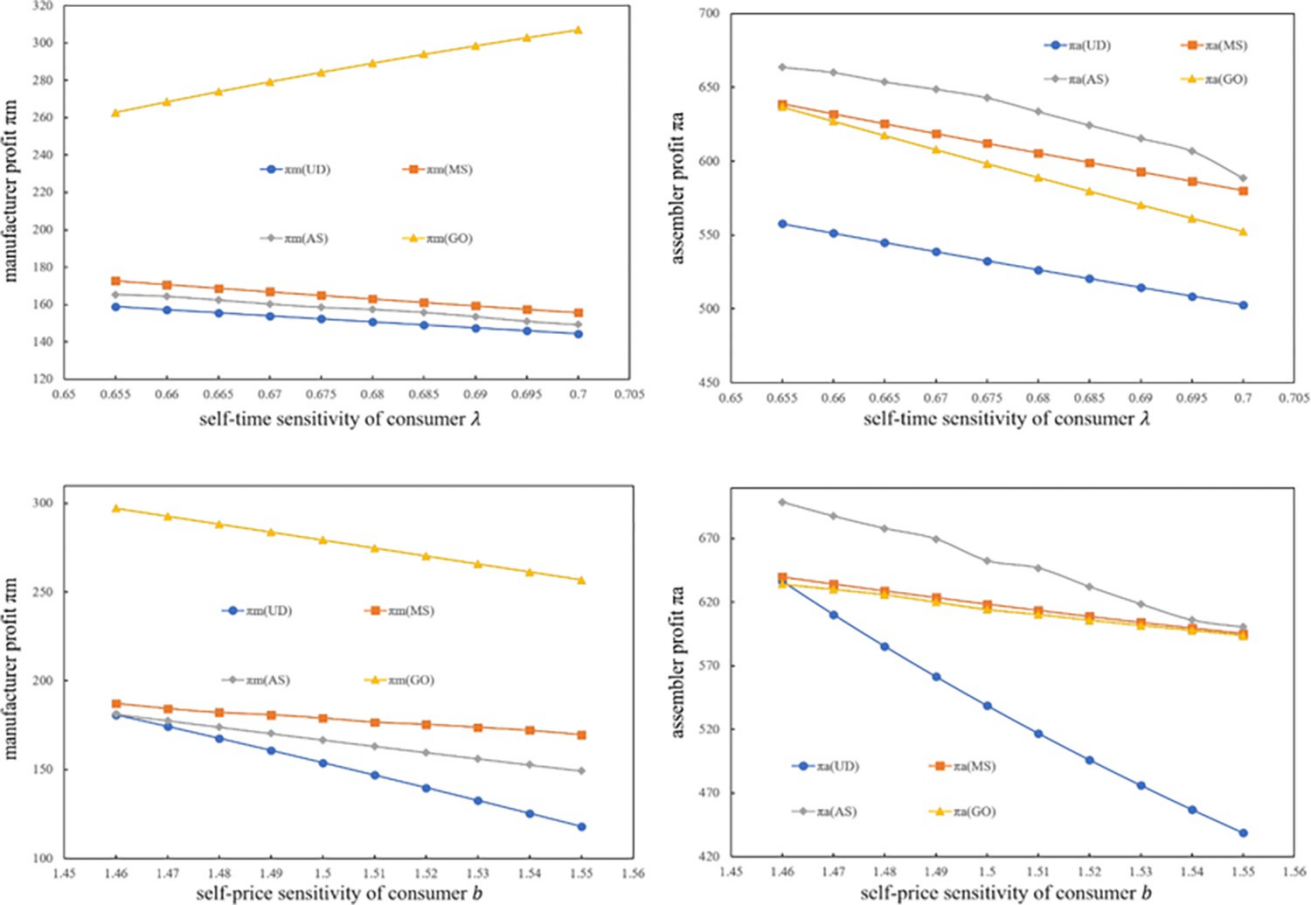
The effect of *b* and *λ* on *π*_*M*_ and *π*_*A*_.

Fig 3 shows the impact of *b* and *λ* on assemblers and manufacturers’ profits. The assembler earns the lowest profit when he or she makes decisions alone. The Stackelberg game led by the assembler can improve his or her own profit, which can decrease rapidly with changes in *b* and *λ*. The assembler’s profit in the MS model is similar to that of the OG model. As *b* and *λ* change, their values change rapidly and become closer. The manufacturer’s profit is low when the assembler makes individual decisions. Stackelberg models are more effective in increasing PCSC profits than the Nash model, especially when the leader is the manufacturer. Moreover, with a reduction in *b* and *λ*, the PCSC profit under the Stackelberg model increases rapidly.

### 6.2. The impact of consumer sensitivity on price and delivery time

**Fig 4:**
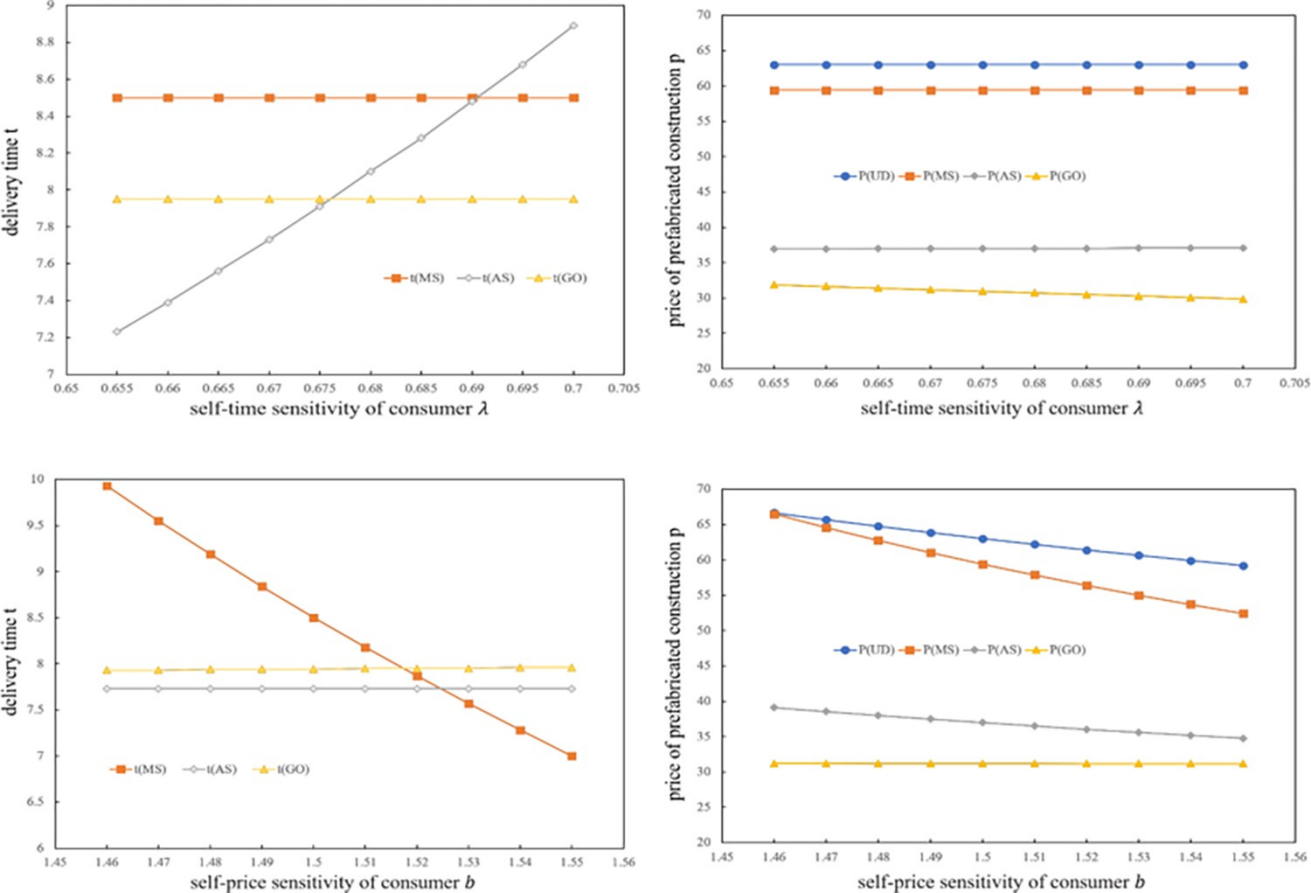
The effect of *b* and *λ* on *t* and *p*.

This subsection investigates the effect of parameters *b* and *λ* on the unit price *p* and delivery time *t*, and the results are shown in Fig 4. The unit price figures show that the lowest value of the PC price is obtained when the global decision is made, followed by the AS game model. The PC price under the unilateral decision model is the highest, which is consistent with the conclusion in Subsection 4.1. From the delivery time figures, it can be seen that in the MS game and OG models, the delivery time *t* is invariable with the change in *λ*. When the assembler leads the Stackelberg game, the delivery time *t* increases rapidly. However, with a change in *b*, the delivery time *t* in the AS and OG models is invariable, and it changes rapidly in the MS game model. A natural way to explain this phenomenon is that when the assembler leads the Stackelberg game, it will ensure its profit by increasing the unit price of the PC first. In addition, the assembler cannot control for the change in the impact of delivery time on consumers. For the same reason, when the manufacturer leads the Stackelberg game, her or she will ensure their profit by reducing the delivery time of nonstandard components first. They also cannot control for the change in the unit price’s impact on consumers. Finally, it can determine that compared to the consumer’s self-time sensitivity *λ*, the consumer’s self-price sensitivity *b* has little impact on the unit price. Additionally, compared to the PC price, the consumer’s self-time sensitivity *λ* has little impact on delivery time.

In comparison with previous conclusions, it finds that the variables that have significant impacts on the manufacturer and assembler are beyond their control. However, they are highly adaptable to the variables that they can control. This indicates that the assembler and manufacturer should carefully consider the consumers’ sensitivity to time and price when making decisions, as both variables have a great influence on their profits.

In general, compared to the UD model, the NG and the two Stackelberg game models effectively increase each party’s profit and that of the entire supply chain. Meanwhile, the OG model can maximize the PCSC profit. Further, the model that the supply chain should use depends on its structure. If the manufacturer plays a leading role in the supply chain, then the manufacturer’s Stackelberg game model should be utilized. If the assembler has more power in the supply chain, then the AS or NG models are preferred. The global optimal model should be chosen when the supply chain members focus on maximizing the supply chain profits.

## 7. Conclusions and future research directions

Constrained by high costs, the development of the PC industry is still at an early stage in many countries. Thus, encouraging initiatives by construction enterprises to produce PCs is key to the development of the industry. However, the stability and efficiency of PCSCs are prerequisites for PCs to demonstrate their advantages. As an important member of the PCSC, the decision of the manufacturer has often been ignored in previous research. It realizes that because of the efficiency of PCs, most consumers are sensitive to the delivery time. Therefore, the production time of the prefabricated components is particularly important. This study investigated the optimal decision in a PCSC under three different power structures. This research then implemented PCSC coordination model with a dynamic wholesale price contract.

The paper has addressed the three research questions including whether the manufacturer adopts the crashing strategy to produce nonstandard components, the optimal strategies of the manufacturer and the assembler under different power structures, and how to solve the conflict between the assembler and the manufacturer, and then provided many other implications. For the first question, the research results showed that under three different power structures, the manufacturer can improve the profits of himself/herself, assemblers, and the entire supply chain by adopting a crash strategy. Therefore, manufacturers should adopt a crash strategy in any competitive environment. For the second question, Section 3 presents an optimal pricing and production strategy of the assembler and manufacturer under three different power structures. In addition, by comparing the optimal strategies with the base model, we found that the manufacturer and assembler strategies are highly related to the initial nonstandard component delivery time *t*_1_ and the wholesale price of prefabricated components *ω*. When *t*_1_ is high and *ω* is low, the manufacturer has sufficient capacity to shorten the production time of non-standard components, and the assembler is willing to lower the price of PCs to attract more consumers. Conversely, the manufacturer and the assembler are constrained with respect to adjusting prices and production times, making it difficult to attract consumers without sacrificing their own profits. For the third question, the dynamic wholesale price model can coordinate PCSCs under three different power structures. This research provides a range of wholesale prices to coordinate the PCSC. However, in this interval, the respective benefits of the assembler and manufacturer are still variable and rely on negotiation between them. A party with a strong negotiating ability can achieve more benefits. Finally, through comparative and numerical analyses, it found that variables such as the consumer sensitivity to time and price have significant impacts on the profits of the manufacturer and assembler, with these variables being beyond their control. Thus, manufacturers and assemblers should seriously consider consumer sensitivity when making decisions.

We summarize the conclusion of the manuscripts into the following five points. (1) Compared with the decentralized decision-making under the three power structures, centralized decision-making can effectively improve the overall profit of the supply chain when the members their own profits and jointly pursue the profit maximization of the supply chain. (2) With the increase of consumer time or price sensitivity, manufacturers and assemblers always need to attract enough consumers by investing in crush costs or reducing construction prices to maintain their own earnings. Therefore, with the increase of consumer sensitivity, the total profit of the supply chain is always decreasing. (3) Under the three power structures, when the assembler or manufacturer leads the supply chain, their own profits are the highest. At the same time, as the party directly selling prefabricated buildings, the profit of the assembler is always higher than that of the manufacturer. (4) The dynamic wholesale price contract can ensure that the profit of the supply chain under decentralized decision-making reaches the profit under centralized decision-making by determining the range of the wholesale price of prefabricated components, and the profit after coordination of each member will not be reduced at least, so as to achieve supply chain coordination. (5) The coordination study gives the range of the coordination coefficient of the wholesale price contract, but the actual value needs to be determined according to the proportion of the rights of the assembler and the manufacturer, and the party with higher rights has the opportunity to greatly improve its profits in the process of supply chain coordination.

Nevertheless, they are highly adaptable to the variables that they can control. This research provides decision-making suggestions for upstream and downstream enterprises of a PCSC in different competitive environments to increase their own profits. This research results can help the PC industry achieve a virtuous cycle and promote the development of PCs.

The future research will extend the scope of this research and enhance its generalizability regarding the following points. First, compared with traditional construction, prefabrication construction is an effective way to reduce carbon emissions, while this paper does not take carbon emission factors into account. Therefore, in the future, it can be further explored that carbon emission decisions of prefabricated construction supply chain members under carbon emission policies. Second, this study examined the deterministic demand of consumers. Therefore, in future research it will consider uncertain consumer demand. Then, the holding cost of the standard components was treated as fixed. However, this may change with the required storage space. In addition, this study considered only one manufacturer and one assembler. However, in real-world scenarios, there are multiple upstream and downstream members in the PCSC. Finally, this study considered only one period. In practice, multiple periods influence PC members’ decisions, such as ordering more standard components in advance to reduce the delivery time of PCs and generate more profit. All of these issues will be addressed in future research.

## Supporting information

S1 Appendix (DOCX)

S1 File (XLSX)
