# Supplementary material for: Modelling of pricing, crashing, and coordination strategies of prefabricated construction supply Chain with power structure
Source: PLoS One. 2023 Aug 10;18(8):e0289630. doi: 10.1371/journal.pone.0289630 (PMC10414617; doi:10.1371/journal.pone.0289630)
Supplement: S1 Appendix — (DOCX) [file pone.0289630.s001.docx]

**Appendix**

**Proof of Proposition 1.** In the unilateral decision model, the objective is to ﬁnd a *p* that maximize the assembler’s proﬁt. It is easy to see that $\pi_{A}^{UD}\left( p \right)$ is a continuous function. Its ﬁrst derivative is:$\frac{\partial\pi_{A}^{UD}\left( p \right)}{\partial p}=ap^{-b}{t_{1}}^{-\lambda}-abp^{-b}{t_{1}}^{-\lambda}+ab\omega p^{-b-1}{t_{1}}^{-\lambda}+abhp^{-b-1}{t_{1}}^{1-\lambda}$, and the second derivative is: $\frac{\partial^{2}\pi_{A}^{UD}\left( p \right)}{\partial p^{2}}=b\left( b-1 \right)ap^{-b-1}{t_{1}}^{-\lambda}-ab(b+1)(\omega+ht_{1})p^{-b-2}{t_{1}}^{-\lambda}$. We assume that $\frac{\partial^{2}\pi_{A}^{UD}\left( p \right)}{\partial p^{2}}<0$, and after simplification we have $p<\frac{(b+1)(\omega+ht_{1}\boldsymbol{)}}{b-1}$. And $p^{UD}=\frac{b(\omega+ht_{1})}{b-1} <\frac{(b+1)(\omega+ht_{1}\boldsymbol{)}}{b-1}.$In this way, $\pi_{A}^{UD}(p)$ is a concave function and the price *p* is always nonnegative, the maximal $\pi_{A}^{UD}(p)$ exist and can be obtained from setting its ﬁrst derivative equals 0. Hence, we have: $p^{UD}=\frac{b(\omega+ht_{1})}{b-1}$. This completes the proof.

**Proof of Proposition 2.** Under manufacturer led Stackelberg game, manufacturer is powerful enough to make decision on the ﬁrst stage. And for assembler’s objective function $\pi_{A}^{SG\left( M \right)}\left( p,t \right)$, the ﬁrst derivative is:$\frac{\partial\pi_{A}^{MS}\left( p,t \right)}{\partial p}=ap^{-b}t^{\boldsymbol{-}\boldsymbol{\lambda}}-(p-\omega-ht)abt^{\boldsymbol{-}\boldsymbol{\lambda}}p^{-b-1}$, and the second derivative is$\frac{\partial^{2}\pi_{A}^{MS}\left( p,t \right)}{\partial p^{2}}=-2abp^{-b-1}t^{-\lambda}+ab(b+1)(p-\omega-ht)t^{-\lambda}p^{-b-2}$. We assume that $\frac{{\partial^{2}\pi}_{A}^{MS}(p,t)}{\partial p^{2}}<0$, and after simplification we have $p<\frac{(b+1)(\omega+ht)}{b-1}$. And $p^{MS}=\frac{b(\omega+ht)}{b-1}<\frac{(b+1)(\omega+ht)}{b-1}$. In this way, $\pi_{A}^{MS}(p,t)$ is a concave function with$p$and the price$p$is always nonnegative, the maximal $\pi_{A}^{MS}(p,t)$ exist and can be obtained from setting its ﬁrst derivative equals 0. Hence, we have: $p^{MS}=\frac{b(\omega+ht)}{b-1}$. And then we substitute $p^{MS}$ into (5), and we get $\pi_{M}^{MS}\left( p^{MS},t \right)=a(\omega-s\left( t_{1}-t \right))\frac{{(b-1)}^{b}}{b^{b}{(\omega+ht)}^{b}t^{\lambda}}$. The ﬁrst derivative is:$\frac{\partial\pi_{M}^{MS}\left( p^{MS},t \right)}{\partial t}=\frac{{a\left( b-1 \right)}^{b}(bh\left( st_{1}+t-\omega\right)-\omega-ht)}{b^{b}{t^{\lambda}(\omega+ht)}^{b+1}}$. The second derivative is:

$$\frac{\partial^{2}\pi_{M}^{MS}\left( p^{MS},t \right)}{\partial t^{2}}=\frac{{aht\left( b-1 \right)}^{b+1}\left( \omega+ht \right)-{a\left( b-1 \right)}^{b}(bh(st_{1}+t-\omega)-\omega-ht)(\lambda\left( \omega+ht \right)+ht\left( b+1 \right))}{b^{b}{t^{\lambda+1}(\omega+ht)}^{b+1}}$$

We assume that $\frac{\partial^{2}\pi_{M}^{MS}\left( p^{SG\left( M \right)},t \right)}{\partial t^{2}}<0$, and after simplification we have $t>\frac{\omega+(b-1)h(\omega-st_{1})}{h(b-1)}$. And we let ﬁrst derivative of $\pi_{A}^{MS}\left( p,t \right)$equals 0, after simplification we have $t^{MS}=\frac{\omega+bh(\omega-st_{1})}{h(b-1)}>\frac{\omega+(b-1)h(\omega-st_{1})}{h(b-1)}$. In this way, $\pi_{A}^{MS}\left( p,t \right)$ is a concave function with $p$ and the price $p$ is always nonnegative, the maximal $\pi_{A}^{MS}\left( p,t \right)$ exist and can be obtained from setting its ﬁrst derivative equals 0. Hence, we have:$t^{MS}=\frac{\omega+bh(\omega-st_{1})}{h(b-1)}$. This completes the proof.

**Proof of Proposition 3.** Under Nash game model, in order to ensure the maximum benefit of both assembler and manufacturer. We calculate the maximum profit separately. For assembler’s objective function $\pi_{A}^{NH}(p,t)$, the ﬁrst derivative is: $\frac{{\partial\pi}_{A}^{NG}(p,t)}{\partial p}=ap^{-b}t^{\boldsymbol{-}\boldsymbol{\lambda}}-(p-\omega-ht)abt^{\boldsymbol{-}\boldsymbol{\lambda}}p^{-b-1}$, and the second derivative is: $\frac{{\partial^{2}\pi}_{A}^{NG}(p,t)}{\partial p^{2}}=-2abp^{-b-1}t^{-\lambda}+ab(b+1)(p-\omega-ht)t^{-\lambda}p^{-b-2}$. We assume that $\frac{{\partial^{2}\pi}_{A}^{NG}(p,t)}{\partial p^{2}}<0$, and after simplification we have $p<\frac{(b+1)(\omega+ht)}{b-1}$. And $p^{NG}=\frac{b(\omega+ht)}{b-1}<\frac{(b+1)(\omega+ht)}{b-1}$. In this way, $\pi_{A}^{NG}(p,t)$ is a concave function with$p$and the price$p$is always nonnegative, the maximal $\pi_{A}^{NG}(p,t)$ exist and can be obtained from setting its ﬁrst derivative equals 0. Hence, we have: $p^{NG}=\frac{b(\omega+ht)}{b-1}$.

For manufacturer’s objective function $\pi_{M}^{NH}(p,t)$, the ﬁrst derivative is: $\frac{{\partial\pi}_{M}^{NG}(p,t)}{\partial t}=ast^{-\lambda}p^{-b}-a\lambda(\omega-s\left( t_{1}-t \right))t^{-\lambda-1}p^{-b}$, and the second derivative is: $\frac{{\partial^{2}\pi}_{M}^{NG}(p,t)}{\partial t^{2}}=-2a\lambda st^{-\lambda-1}p^{-b}+a\lambda(\lambda+1)\left( \omega-s(t_{1}-t) \right)t^{-\lambda-2}p^{-b}$. We assume that $\frac{{\partial^{2}\pi}_{M}^{NG}(p,t)}{\partial t^{2}}<0$, and after simplification we have $t<\frac{(\lambda+1)(st_{1}-\omega)}{\lambda s-s}$. And $t^{NG}=\frac{\lambda(st_{1}-\omega)}{\lambda s-s}<\frac{(\lambda+1)(st_{1}-\omega)}{\lambda s-s}$. In this way, $\pi_{M}^{NG}(p,t)$ is a concave function with *t* and the crash time *t* is always nonnegative, the maximal $\pi_{M}^{NG}(p,t)$ exist and can be obtained from setting its ﬁrst derivative equals 0. Hence, we have: $t^{NG}=\frac{\lambda(st_{1}-\omega)}{\lambda s-s}$. This completes the proof.

**Proof of Proposition 4.** Under assembler led Stackelberg game, assembler is powerful enough to make decision on the ﬁrst stage. And for manufacturer’s objective function $\pi_{M}^{AS}\left( p,t \right)$, the ﬁrst derivative is: $\frac{\partial\pi_{M}^{AS}\left( p,t \right)}{\partial t}=ast^{-\lambda}p^{-b}-a\lambda(\omega-s\left( t_{1}-t \right))t^{-\lambda-1}p^{-b}$, and the second derivative is$\frac{\partial^{2}\pi_{M}^{AS}\left( p,t \right)}{\partial t^{2}}=-2a\lambda st^{-\lambda-1}p^{-b}+a\lambda(\lambda+1)\left( \omega-s(t_{1}-t) \right)t^{-\lambda-2}p^{-b}$. We assume that $\frac{{\partial^{2}\pi}_{M}^{AS}(p,t)}{\partial t^{2}}<0$, and after simplification we have $t<\frac{(\lambda+1)(st_{1}-\omega)}{\lambda s-s}$. And $t^{AS}=\frac{\lambda(st_{1}-\omega)}{\lambda s-s}<\frac{(\lambda+1)(st_{1}-\omega)}{\lambda s-s}$. In this way, $\pi_{M}^{AS}(p,t)$ is a concave function with *t* and the crash time *t* is always nonnegative, the maximal $\pi_{M}^{AS}(p,t)$ exist and can be obtained from setting its ﬁrst derivative equals 0. Hence, we have: $t^{AS}=\frac{\lambda(st_{1}-\omega)}{\lambda s-s}$.

And then we substitute $t^{AS}$ into (11), and we get $\pi_{A}^{AS}\left( p,t^{SG(A)} \right)=ap^{-b}(p-\omega-\frac{h\lambda\left( st_{1}-\omega\right)}{\lambda s-s}){(\frac{\lambda s-s}{\lambda\left( st_{1}-\omega\right)})}^{\lambda}$. The ﬁrst derivative is: $\frac{\partial\pi_{A}^{AS}\left( p,t^{AS} \right)}{\partial p}=ap^{-b}\frac{s^{\lambda}{(\lambda-1)}^{\lambda}}{\lambda^{\lambda}{(st_{1}-\omega)}^{\lambda}}-a{bp}^{-b-1}\frac{s^{\lambda}\left( \lambda-1 \right)^{\lambda}}{\lambda^{\lambda}\left( st_{1}-\omega\right)^{\lambda}}(p-\omega-\frac{h\lambda\left( st_{1}-\omega\right)}{\lambda s-s})$. The second derivative is:$\frac{\partial^{2}\pi_{A}^{AS}\left( p,t^{AS} \right)}{\partial p^{2}}=\frac{s^{\lambda}\left( \lambda-1 \right)^{\lambda}}{\lambda^{\lambda}\left( st_{1}-\omega\right)^{\lambda}}(a{b\left( b+1 \right)p}^{-b-2}(p-\omega-\frac{h\lambda\left( st_{1}-\omega\right)}{\lambda s-s})-2abp^{-b-1})$. We assume that $\frac{\partial^{2}\pi_{A}^{AS}\left( p,t^{AS} \right)}{\partial p^{2}}<0$, and after simplification we have $p<\frac{b+1}{b-1}(\omega+\frac{h\lambda\left( st_{1}-\omega\right)}{\lambda s-s})$. And $p^{AS}=\frac{b}{b-1}\left( \omega+\frac{h\lambda\left( st_{1}-\omega\right)}{\lambda s-s} \right)<\frac{b+1}{b-1}(\omega+\frac{h\lambda\left( st_{1}-\omega\right)}{\lambda s-s})$.In this way, $\pi_{A}^{AS}\left( p,t \right)$ is a concave function with *p* and the price *p* is always nonnegative, the maximal $\pi_{A}^{AS}\left( p,t \right)$ exist and can be obtained from setting its ﬁrst derivative equals 0. Hence, we have: $p^{AS}=\frac{b}{b-1}\left( \omega+\frac{h\lambda\left( st_{1}-\omega\right)}{\lambda s-s} \right)$. This completes the proof.

**Proof of Proposition 5.** To make the crashing strategy works, the first priority is to ensure that the nonstandard components’ production time is shorter than the initial production time ($t\leq t_{1}$). From proposition 2, we have $t^{MS}=\frac{\omega+bh(\omega-st_{1})}{h(b-1)}$, and after calculating $\frac{\omega+bh(\omega-st_{1})}{h(b-1)}<t_{1}$, we have $t_{1}\geq\frac{\omega(bh+1)}{h(bs+b-1)}$.

From proposition 1 and the proof of proposition 2, we have $p^{UD}=\frac{b(\omega+ht_{1})}{b-1} \mathrm{and} p^{MS}=\frac{b(\omega+ht^{MS})}{b-1}$.

And the different is only on producing time $t$. Then we set a function $p(t)=\frac{b(\omega+ht)}{b-1}$. And $\frac{\partial p(t)}{\partial t}=\frac{bh}{b-1}$, which means $p\left( t \right)$ is a monotonically increasing function of $t$. When $\frac{\omega(bh+1)}{h(bs+b-1)}<t_{1}$ , we have $p^{MS}<p^{UD}$.

From proposition 1 and the proof of proposition 2, we substitute $p^{UD}$($p^{MS}$) into $\pi_{A}^{UD}$($\pi_{A}^{MS}$), and we have $\pi_{A}^{UD}(t)=\frac{a}{b}{(\frac{b-1}{b(\omega+ht_{1})})}^{b-1}{t_{1}}^{-\lambda}$ and $\pi_{A}^{MS}\left( t^{MS} \right)=\frac{a}{b}{(\frac{b-1}{b(\omega+ht^{MS})})}^{b-1}{t^{MS}}^{-\lambda}$. And the different is only on producing time $t$. Then we set a function $M\left( t \right)=\frac{a}{b}{(\frac{b-1}{b(\omega+ht)})}^{b-1}t^{-\lambda} \mathrm{and}$ $\frac{\partial M\left( t \right)}{\partial t}=-\frac{a}{b}\left( \frac{b-1}{b\left( \omega+ht \right)} \right)^{b-1}(\frac{\left( b-1 \right)bh}{b\left( \omega+ht \right)t^{\lambda}}+\lambda t^{-\lambda-1})<0$, which means $M\left( t \right)$ is a monotonically increasing function of $t$. We have $\pi_{A}^{MS}>\pi_{A}^{UD}$ when $t_{1}\geq\frac{\omega(bh+1)}{h(bs+b-1)}$.

From proposition 1 and the proof of proposition 2, we substitute $p^{UD}$($p^{MS}$) into $\pi_{M}^{UD}$($\pi_{M}^{MS}$), and we have $\frac{\pi_{M}^{MS}\left( t^{MS} \right)}{\pi_{M}^{UD}(t)}=\frac{(\omega-st_{1}-st){(b\left( \omega+ht_{1} \right))}^{b}{t_{1}}^{\lambda}}{\omega{(b\left( \omega+ht^{MS} \right))}^{b}{t^{MS}}^{\lambda}}$. It’s can be seen that the magnitude of $\pi_{M}^{MS}$ and $\pi_{M}^{UD}$ depends on $t$. And when $t_{1}\geq\frac{\omega(bh+1)}{h(bs+b-1)}$, we have $\pi_{M}^{MS}>\pi_{M}^{UD}$. In the same way $\frac{\pi^{MS}}{\pi^{UD}}=\frac{(b\omega+ht^{MS}+s(b-1)(t^{MS}-t_{1})){(b\left( \omega+ht_{1} \right))}^{b}{t_{1}}^{\lambda}}{(b\omega+ht_{1}){(b\left( \omega+ht^{MS} \right))}^{b}{t^{MS}}^{\lambda}}$. It’s can be seen that the magnitude of $\pi^{MS}$ and $\pi^{UD}$ depends on $t$. And when $t_{1}\geq\frac{\omega(bh+1)}{h(bs+b-1)}$, we have $\pi^{MS}>\pi^{UD}$. This completes the proof.

**Proof of Proposition 6.** To make the crashing strategy works, the first priority is to ensure that the producing time of nonstandard is shorter than the initial production time ($t\leq t_{1}$). From proposition 4, we have $t^{NG}=\frac{\lambda(st_{1}-\omega)}{\lambda s-s}$, and after calculating $\frac{\lambda(st_{1}-\omega)}{\lambda s-s}<t_{1}$, we have $t_{1}\geq\frac{\lambda\omega}{s}$.

From proposition 1 and the proof of proposition 4, we have $p^{UD}=\frac{b(\omega+ht_{1})}{b-1} \mathrm{and} p^{NG}=\frac{b(\omega+ht^{NG})}{b-1}$. And the different is only on producing time $t$. Then we set a function $p(t)=\frac{b(\omega+ht)}{b-1}$. And $\frac{\partial p(t)}{\partial t}=\frac{bh}{b-1}$, which means $p\left( t \right)$ is a monotonically increasing function of $t$. When $\frac{\lambda\omega}{s}<t_{1}$ , we have $p^{NG}<p^{UD}$.

From proposition 1 and the proof of proposition 4, we substitute $p^{UD}$($p^{NG}$) into $\pi_{A}^{UD}$($\pi_{A}^{NG}$), and we have $\pi_{A}^{UD}=\frac{a}{b}{(\frac{b-1}{b(\omega+ht_{1})})}^{b-1}{t_{1}}^{-\lambda}$ and $\pi_{A}^{NG}\left( t^{NG} \right)=\frac{a}{b}{(\frac{b-1}{b(\omega+ht^{NG})})}^{b-1}{t^{NG}}^{-\lambda}$. And the different is only on producing time $t$. Then we set a function $M\left( t \right)=\frac{a}{b}{(\frac{b-1}{b(\omega+ht)})}^{b-1}t^{-\lambda} \mathrm{and}$ $\frac{\partial M\left( t \right)}{\partial t}=-\frac{a}{b}\left( \frac{b-1}{b\left( \omega+ht \right)} \right)^{b-1}(\frac{\left( b-1 \right)bh}{b\left( \omega+ht \right)t^{\lambda}}+\lambda t^{-\lambda-1})<0$, which means $M\left( t \right)$ is a monotonically decreasing function of $t$. We have $\pi_{A}^{NG}\geq\pi_{A}^{UD}$ when $t_{1}\geq\frac{\lambda\omega}{s}$.

From proposition 1 and the proof of proposition 4, we substitute $p^{NG}$($p^{MS}$) into $\pi_{M}^{NG}$($\pi_{M}^{MS}$), and we have $\frac{\pi_{M}^{NG}}{\pi_{M}^{UD}}=\frac{(\omega-st_{1}-st^{NG}){(b\left( \omega+ht_{1} \right))}^{b}{t_{1}}^{\lambda}}{\omega{(b\left( \omega+ht^{NG} \right))}^{b}{t^{NG}}^{\lambda}}$. It’s can be seen that the magnitude of $\pi_{M}^{NG}$ and $\pi_{M}^{UD}$ depends on $t$. And when $t_{1}\geq\frac{\lambda\omega}{s}$, we have $\pi_{M}^{NG}>\pi_{M}^{UD}$. In the same way $\frac{\pi^{NG}}{\pi^{UD}}=\frac{(b\omega+ht^{NG}+s(b-1)(t^{NG}-t_{1})){(b\left( \omega+ht_{1} \right))}^{b}{t_{1}}^{\lambda}}{(b\omega+ht_{1}){(b\left( \omega+ht^{NG} \right))}^{b}{t^{NG}}^{\lambda}}$. It’s can be seen that the magnitude of $\pi^{MS}$ and $\pi^{UD}$ depends on $t$. And when $t_{1}\geq\frac{\lambda\omega}{s}$, we have $\pi^{MS}\geq\pi^{UD}$. This completes the proof.

**Proof of Proposition 7.** From proposition 3 and 4, $p^{AS}=p^{NG}$ and $t^{AS}=t^{NG}$. Then it's easy to figure out that $\pi_{A}^{AS}=\pi_{A}^{NG}$, $\pi_{M}^{AS}=\pi_{M}^{NG}$ and $\pi^{NG}=\pi^{UD}$. Which means that the conclusion from proposition 5 is also apply to Assembler Stackelberg game. This completes the proof.

**Proof of Proposition 8.** We find out that the optimal decision of assemblers and manufacturers is same in Nash game model and Assembler Stackelberg model. Then we only compare the decision of Assembler Stackelberg model and Manufacturer Stackelberg model in this part.

From proposition 2 and 4, we have $t^{MS}=\frac{\omega+bh(\omega-st_{1})}{h(b-1)}$ and $t^{AS}=\frac{\lambda(st_{1}-\omega)}{\lambda s-s}$. First of all, we calculate $t^{AS}>t^{MS}$, and $\frac{t^{AS}}{t^{MS}}=\frac{\lambda h(st_{1}-\omega)(b-1)}{\omega s\left( \lambda-1 \right)+bhs\left( \lambda-1 \right)(\omega-st_{1})}>1$. From the proof of proposition 2 and 6, we have $p^{MS}=\frac{b(\omega+ht^{MS})}{b-1} \mathrm{and} p^{AS}=\frac{b(\omega+ht^{AS})}{b-1}$. And the different is only on producing time $t$. Then we set a function $p(t)=\frac{b(\omega+ht)}{b-1}$. And $\frac{\partial p(t)}{\partial t}=\frac{bh}{b-1}$, which means $p\left( t \right)$ is a monotonically increasing function of $t$. When $\frac{t^{AS}}{t^{MS}}=\frac{\lambda h(st_{1}-\omega)(b-1)}{\omega s\left( \lambda-1 \right)+bhs\left( \lambda-1 \right)(\omega-st_{1})}>1$ , we have $p^{MS}<p^{AS}$.

From the proof of proposition 2 and 4, we substitute $p^{AS}$($p^{MS}$) into $\pi_{A}^{AS}$($\pi_{A}^{MS}$), and we have $\pi_{A}^{AS}\left( t^{AS} \right)=\frac{a}{b}{(\frac{b-1}{b(\omega+ht^{AS})})}^{b-1}{t^{AS}}^{-\lambda}$ and $\pi_{A}^{MS}\left( t^{MS} \right)=\frac{a}{b}{(\frac{b-1}{b(\omega+ht^{MS})})}^{b-1}{t^{MS}}^{-\lambda}$. And the different is only on producing time $t$. Then we set a function $M\left( t \right)=\frac{a}{b}{(\frac{b-1}{b(\omega+ht)})}^{b-1}t^{-\lambda} \mathrm{and}$ $\frac{\partial M\left( t \right)}{\partial t}=-\frac{a}{b}\left( \frac{b-1}{b\left( \omega+ht \right)} \right)^{b-1}(\frac{\left( b-1 \right)bh}{b\left( \omega+ht \right)t^{\lambda}}+\lambda t^{-\lambda-1})<0$, which means $M\left( t \right)$ is a monotonically increasing function of $t$. We have $\pi_{A}^{AS}<\pi_{A}^{MS}$ when $\frac{t^{AS}}{t^{MS}}=\frac{\lambda h(st_{1}-\omega)(b-1)}{\omega s\left( \lambda-1 \right)+bhs\left( \lambda-1 \right)(\omega-st_{1})}>1$.

From the proof of proposition 2 and 4, we substitute $p^{AS}$($p^{MS}$) into $\pi_{M}^{AS}$($\pi_{M}^{MS}$), and we have $\frac{\pi_{M}^{AS}}{\pi_{M}^{MS}}=\frac{(\omega-st_{1}-st^{AS}){(b\left( \omega+ht^{MS} \right))}^{b}{t^{MS}}^{\lambda}}{(\omega-st_{1}-st^{MS}){(b\left( \omega+ht^{AS} \right))}^{b}{t^{AS}}^{\lambda}}$. It’s can be seen that the magnitude of $\pi_{M}^{NG}$ and $\pi_{M}^{UD}$ depends on $t$. And when $\frac{t^{AS}}{t^{MS}}=\frac{\lambda h(st_{1}-\omega)(b-1)}{\omega s\left( \lambda-1 \right)+bhs\left( \lambda-1 \right)(\omega-st_{1})}>1$, we have $\pi_{M}^{AS}<\pi_{M}^{MS}$. In the same way$\frac{\pi^{AS}}{\pi^{MS}}=\frac{(b\omega+ht^{AS}+s(b-1)(t^{MS}-t_{1})){(b\left( \omega+ht^{MS} \right))}^{b}{t^{MS}}^{\lambda}}{(b\omega+ht^{MS}+s(b-1)(t^{AS}-t_{1})){(b\left( \omega+ht^{AS} \right))}^{b}{t^{AS}}^{\lambda}}$. It’s can be seen that the magnitude of $\pi^{MS}$ and $\pi^{UD}$ depends on $t$. And when $\frac{t^{AS}}{t^{MS}}=\frac{\lambda h(st_{1}-\omega)(b-1)}{\omega s\left( \lambda-1 \right)+bhs\left( \lambda-1 \right)(\omega-st_{1})}>1$, we have $\pi^{AS}<\pi^{MS}$. This completes the proof.

**Proof of Proposition 9.** In the optimal global model, the objective is to ﬁnd a $p$ and a $t$ that maximize the whole supply chain’s proﬁt. For $\pi^{GO}\left( p,t \right)$, the ﬁrst derivative is: $\frac{\partial\pi^{GO}\left( p,t \right)}{\partial p}=\left( 1-b \right)ap^{-b}t^{-\lambda}+ab(st_{1}+\left( h-s \right)t)p^{-b-1}t^{-\lambda}$ and $\frac{\partial\pi^{GO}\left( p,t \right)}{\partial t}=-\lambda ap^{-b+1}t^{-\lambda-1}+\lambda st_{1}ap^{-b}t^{-\lambda-1}+(\lambda-1)(h-s)ap^{-b}t^{-\lambda}$, and the second derivative is: $\frac{\partial^{2}\pi^{GO}\left( p,t \right)}{\partial p^{2}}=b\left( b-1 \right)ap^{-b-1}t^{-\lambda}-ab\left( b+1 \right)\left( st_{1}+\left( h-s \right)t \right)p^{-b-2}t^{-\lambda}<0$, $\frac{\partial^{2}\pi^{GO}\left( p,t \right)}{\partial t^{2}}=a\lambda\left( \lambda+1 \right)t^{-\lambda-2}\left( p^{-b+1}-p^{-b}st_{1} \right)+a\lambda\left( \lambda-1 \right)\left( s-h \right)p^{-b}t^{-\lambda-1}<0$, $\frac{\partial^{2}\pi^{GO}\left( p,t \right)}{\partial p\partial t}=\lambda\left( b-1 \right)ap^{-b}t^{-\lambda-1}-ab\left( s-h \right)p^{-b-1}t^{-\lambda}-ab\lambda\left( st_{1}+\left( h-s \right)t \right)p^{-b-1}t^{-\lambda-1}<0$ and $\frac{\partial^{2}\pi^{GO}\left( p,t \right)}{\partial t\partial p}=a\lambda t^{-\lambda-1}\left( \left( b+1 \right)p^{-b-1}-bst_{1}p^{-b-1} \right)+ab\left( \lambda-1 \right)\left( s-h \right)p^{-b-1}t^{-\lambda}<0$.

After calculation, we obtain $\left| \begin{matrix} \frac{\partial^{2}\pi^{GO}\left( p,t \right)}{\partial p^{2}} & \frac{\partial^{2}\pi^{GO}(p,t)}{\partial p\partial t} \\ \frac{\partial^{2}\pi^{GO}(p,t)}{\partial t\partial p} & \frac{\partial^{2}\pi^{GO}(p,t)}{\partial t^{2}} \end{matrix} \right|$=$\frac{\partial^{2}\pi^{GO}\left( p,t \right)}{\partial p^{2}}\frac{\partial^{2}\pi^{GO}(p,t)}{\partial t^{2}}-\frac{\partial^{2}\pi^{GO}(p,t)}{\partial p\partial t}\frac{\partial^{2}\pi^{GO}(p,t)}{\partial t\partial p}$. And $\frac{b\left( b-1 \right)pt-b\left( b+1 \right)\left( st_{1}+\left( h-s \right)t \right)t}{\lambda\left( b-1 \right)p^{2}-b\left( s-h \right)pt^{-\lambda}-b\lambda\left( st_{1}+\left( h-s \right)t \right)p}={\frac{\partial^{2}\pi^{GO}\left( p,t \right)}{\partial p^{2}}}/{\frac{\partial^{2}\pi^{GO}(p,t)}{\partial p\partial t}}>0$, and $\frac{\lambda\left( \lambda+1 \right)\left( p^{2}-pst_{1} \right)+\lambda\left( \lambda-1 \right)\left( s-h \right)pt}{\lambda t\left( \left( b+1 \right)-bst_{1} \right)+b\left( \lambda-1 \right)\left( s-h \right)t^{2}}={\frac{\partial^{2}\pi^{GO}(p,t)}{\partial t^{2}}}/{\frac{\partial^{2}\pi^{GO}(p,t)}{\partial t\partial p}}$. Thus, we have $\left| \begin{matrix} \frac{\partial^{2}\pi^{GO}\left( p,t \right)}{\partial p^{2}} & \frac{\partial^{2}\pi^{GO}(p,t)}{\partial p\partial t} \\ \frac{\partial^{2}\pi^{GO}(p,t)}{\partial t\partial p} & \frac{\partial^{2}\pi^{GO}(p,t)}{\partial t^{2}} \end{matrix} \right|>0$**.** Then, we can get that $\pi^{GO}\left( p,t \right)$ is jointly concave in $p$ and $t$. And we obtain the optimal profit of supply chain by plugging optimal $p$ and $t$ into assembler’s profit equation. This completes the proof.

**Proof of Proposition 10.** We only compare the magnitude of $\pi^{GO}$, $\pi^{AS}$ and $\pi^{MS}$ because of that the results of Nash Game model are same as Assembler Stackelberg model. From proof of proposition 9, we have that $\pi^{GO}\left( p,t \right)$ is jointly concave in $p$ and $t$. And the optimal delivery time of nonstandard components and price of prefabricated construction is $p^{GO} \mathrm{and} t^{GO}$. Then we have conclusion that any other price and delivery time will not higher than $\pi^{GO}$. Thus, we conclude that $\pi^{GO}>\pi^{AS} and \pi^{GO}>\pi^{MS}$. This completes the proof.

**Proof of Proposition 11.** In order to achieve coordination, the first goal is to guarantee that the optimal profit of supply chain can reach that in optimal global model ($\pi^{CMS}=\pi^{GO}$). And it’s hard to compare the price of two models directly. The profit of the whole supply chain is related to the price ($p$) and the delivery time ($t$) of prefabricated construction. So, we just need to ensure that the optimal price and delivery time in coordination model can reach that in optimal global model. Then we can prove that the profit of supply chain under coordination model can reach that in optimal global model. We substitute $p^{GO}$ and $t^{GO}$ into formula (15) and (16), and we have: $\pi_{A}^{CMS}\left( p^{GO},t^{GO} \right)=\frac{a(bst_{1}\left( s-h \right)-c_{0}\left( b+\lambda-1 \right)\left( s-h \right)-h\lambda st_{1})\left( b+\lambda-1 \right)^{b+\lambda-1}{(s-h)}^{\lambda-1}}{b^{b}\lambda^{\lambda}s^{b+\lambda}{t_{1}}^{b+\lambda}}-\theta$ and $\pi_{M}^{CMS}\left( p^{GO},t^{GO} \right)=\frac{a(c_{0}\left( b+\lambda-1 \right)\left( s-h \right)-s\left( b-1 \right)\left( s-h \right)t_{1}+h\lambda st_{1})\left( b+\lambda-1 \right)^{b+\lambda-1}{(s-h)}^{\lambda-1}}{b^{b}\lambda^{\lambda}s^{b+\lambda}{t_{1}}^{b+\lambda}}+\theta$. To ensure the coordination strategy working, we have to guarantee that $\pi_{A}^{CMS}\left( p^{GO},t^{GO} \right)-\pi_{A}^{MS}=\frac{a\left( bst_{1}\left( s-h \right)-c_{0}\left( b+\lambda-1 \right)\left( s-h \right)-h\lambda st_{1} \right)\left( b+\lambda-1 \right)^{b+\lambda-1}\left( s-h \right)^{\lambda-1}}{b^{b}\lambda^{\lambda}s^{b+\lambda}{t_{1}}^{b+\lambda}}-\frac{ah^{\lambda}\left( b-1 \right)^{\lambda+2b-2}}{b^{b}\left( b\omega+bh\left( \omega-st_{1} \right) \right)^{b-1}\left( c_{0}+bh\left( \omega-st_{1} \right) \right)^{\lambda}}-\theta\geq0$ and $\pi_{M}^{CMS}\left( p^{GO},t^{GO} \right)-\pi_{M}^{MS}=\frac{a\left( c_{0}\left( b+\lambda-1 \right)\left( s-h \right)-s\left( b-1 \right)\left( s-h \right)t_{1}+h\lambda st_{1} \right)\left( b+\lambda-1 \right)^{b+\lambda-1}\left( s-h \right)^{\lambda-1}}{b^{b}\lambda^{\lambda}s^{b+\lambda}{t_{1}}^{b+\lambda}}-\frac{ah^{\lambda-1}\left( b-1 \right)^{\lambda+2b-1}\left( \omega h\left( b-1 \right)-sht_{1}\left( b-1+bs \right)+s\omega\left( bh+1 \right) \right)}{b^{b}\left( b\omega+bh\left( \omega-st_{1} \right) \right)^{b}\left( \omega+bh\left( \omega-st_{1} \right) \right)^{\lambda}}+\theta\geq0$. Thus, we have the range of $\theta^{1}$: $\theta^{1}\in\left[ \theta_{min}^{1}, \theta_{max}^{1} \right]$ to guarantee that the optimal profit of supply chain can reach that in optimal global model.

$$\theta_{min}^{1}=\frac{ah^{\lambda-1}\left( b-1 \right)^{\lambda+2b-1}\left( \omega h\left( b-1 \right)-sht_{1}\left( b-1+bs \right)+s\omega\left( bh+1 \right) \right)}{b^{b}\left( b\omega+bh\left( \omega-st_{1} \right) \right)^{b}\left( \omega+bh\left( \omega-st_{1} \right) \right)^{\lambda}}-\frac{a\left( c_{0}\left( b+\lambda-1 \right)\left( s-h \right)-s\left( b-1 \right)\left( s-h \right)t_{1}+h\lambda st_{1} \right)\left( b+\lambda-1 \right)^{b+\lambda-1}\left( s-h \right)^{\lambda-1}}{b^{b}\lambda^{\lambda}s^{b+\lambda}{t_{1}}^{b+\lambda}}$$

$$\theta_{max}^{1}=\frac{a\left( bst_{1}\left( s-h \right)-c_{0}\left( b+\lambda-1 \right)\left( s-h \right)-h\lambda st_{1} \right)\left( b+\lambda-1 \right)^{b+\lambda-1}\left( s-h \right)^{\lambda-1}b^{b}\left( b\omega+bh\left( \omega-st_{1} \right) \right)^{b-1}\left( c_{0}+bh\left( \omega-st_{1} \right) \right)^{\lambda}}{b^{2b}\lambda^{\lambda}s^{b+\lambda}{t_{1}}^{b+\lambda}\left( b\omega+bh\left( \omega-st_{1} \right) \right)^{b-1}\left( c_{0}+bh\left( \omega-st_{1} \right) \right)^{\lambda}}-\frac{-ah^{\lambda}\left( b-1 \right)^{\lambda+2b-2}b^{b}\lambda^{\lambda}s^{b+\lambda}{t_{1}}^{b+\lambda}}{b^{2b}\lambda^{\lambda}s^{b+\lambda}{t_{1}}^{b+\lambda}\left( b\omega+bh\left( \omega-st_{1} \right) \right)^{b-1}\left( c_{0}+bh\left( \omega-st_{1} \right) \right)^{\lambda}}$$

After the coordination of dynamic wholesale price mechanism, it is necessary to ensure that the profit of assemblers and manufacturers is not less than that before negotiation, $\pi_{A}^{CMS}-\pi_{A}^{MS}=\frac{ah^{\lambda}\left( b-1 \right)^{\lambda+2b-2}}{b^{b}\left( bc_{0}+bh\left( c_{0}-st_{1} \right) \right)^{b-1}\left( c_{0}+bh\left( c_{0}-st_{1} \right) \right)^{\lambda}}-\frac{ah^{\lambda}\left( b-1 \right)^{\lambda+2b-2}}{b^{b}\left( b\omega+bh\left( \omega-st_{1} \right) \right)^{b-1}\left( c_{0}+bh\left( \omega-st_{1} \right) \right)^{\lambda}}-\theta\geq0$ and $\pi_{M}^{CMS}-\pi_{M}^{MS}=\frac{ah^{\lambda-1}\left( b-1 \right)^{\lambda+2b-1}\left( c_{0}h\left( b-1 \right)-sht_{1}\left( b-1+bs \right)+sc_{0}\left( bh+1 \right) \right)}{b^{b}\left( bc_{0}+bh\left( c_{0}-st_{1} \right) \right)^{b}\left( c_{0}+bh\left( c_{0}-st_{1} \right) \right)^{\lambda}}-\frac{ah^{\lambda-1}\left( b-1 \right)^{\lambda+2b-1}\left( \omega h\left( b-1 \right)-sht_{1}\left( b-1+bs \right)+s\omega\left( bh+1 \right) \right)}{b^{b}\left( b\omega+bh\left( \omega-st_{1} \right) \right)^{b}\left( \omega+bh\left( \omega-st_{1} \right) \right)^{\lambda}}+\theta\geq0$. Then, we can determine the range of $\theta^{2}$: $\theta^{2}\in\left[ \theta_{min}^{2}, \theta_{max}^{2} \right]$.

$$\theta_{min}^{2}=\frac{ah^{\lambda-1}\left( b-1 \right)^{\lambda+2b-1}(\omega h\left( b-1 \right)-sht_{1}\left( b-1+bs \right)+s\omega\left( bh+1 \right))}{b^{b}{(b\omega+bh(\omega-st_{1}))}^{b}{(\omega+bh(\omega-st_{1}))}^{\lambda}}-\frac{ah^{\lambda-1}\left( b-1 \right)^{\lambda+2b-1}(c_{0}h\left( b-1 \right)-sht_{1}\left( b-1+bs \right)+sc_{0}\left( bh+1 \right))}{b^{b}{(bc_{0}+bh(c_{0}-st_{1}))}^{b}{(c_{0}+bh(c_{0}-st_{1}))}^{\lambda}}$$

$$\theta_{max}^{2}=\frac{ah^{\lambda}\left( b-1 \right)^{\lambda+2b-2}(\left( b\omega+bh\left( \omega-st_{1} \right) \right)^{b-1}\left( \omega+bh\left( \omega-st_{1} \right) \right)^{\lambda}-\left( bc_{0}+bh\left( c_{0}-st_{1} \right) \right)^{b-1}\left( c_{0}+bh\left( c_{0}-st_{1} \right) \right)^{\lambda})}{b^{b}{(bc_{0}+bh(c_{0}-st_{1}))}^{b-1}{(c_{0}+bh(c_{0}-st_{1}))}^{\lambda}{(b\omega+bh(\omega-st_{1}))}^{b-1}{(\omega+bh(\omega-st_{1}))}^{\lambda}}$$

The range of $\theta^{1}$ ensure that the profit of supply chain under Manufacturer Stackelberg game with dynamic whole sale price contract can reach that profit under optimal global model. And the range of $\theta^{2}$ guarantee that the profit of Assembler and Manufacturer won’t lower than before after coordination. To ensure the success of the coordination strategy, we need to ensure that both conditions are met. And we take the intersection of $\theta^{1}$ and $\theta^{2}$. Then we have the range of $\theta$: $\theta\in\left[ \theta_{min}, \theta_{max} \right]$.

$$\theta_{min}=\frac{ah^{\lambda-1}\left( b-1 \right)^{\lambda+2b-1}\left( \omega h\left( b-1 \right)-sht_{1}\left( b-1+bs \right)+s\omega\left( bh+1 \right) \right)}{b^{b}\left( b\omega+bh\left( \omega-st_{1} \right) \right)^{b}\left( \omega+bh\left( \omega-st_{1} \right) \right)^{\lambda}}-\frac{a\left( c_{0}\left( b+\lambda-1 \right)\left( s-h \right)-s\left( b-1 \right)\left( s-h \right)t_{1}+h\lambda st_{1} \right)\left( b+\lambda-1 \right)^{b+\lambda-1}\left( s-h \right)^{\lambda-1}}{b^{b}\lambda^{\lambda}s^{b+\lambda}{t_{1}}^{b+\lambda}}$$

$$\theta_{max}=\frac{a\left( bst_{1}\left( s-h \right)-c_{0}\left( b+\lambda-1 \right)\left( s-h \right)-h\lambda st_{1} \right)\left( b+\lambda-1 \right)^{b+\lambda-1}\left( s-h \right)^{\lambda-1}b^{b}\left( b\omega+bh\left( \omega-st_{1} \right) \right)^{b-1}\left( c_{0}+bh\left( \omega-st_{1} \right) \right)^{\lambda}}{b^{2b}\lambda^{\lambda}s^{b+\lambda}{t_{1}}^{b+\lambda}\left( b\omega+bh\left( \omega-st_{1} \right) \right)^{b-1}\left( c_{0}+bh\left( \omega-st_{1} \right) \right)^{\lambda}}-\frac{-ah^{\lambda}\left( b-1 \right)^{\lambda+2b-2}b^{b}\lambda^{\lambda}s^{b+\lambda}{t_{1}}^{b+\lambda}}{b^{2b}\lambda^{\lambda}s^{b+\lambda}{t_{1}}^{b+\lambda}\left( b\omega+bh\left( \omega-st_{1} \right) \right)^{b-1}\left( c_{0}+bh\left( \omega-st_{1} \right) \right)^{\lambda}}$$

Thus, the dynamic wholesale price contract can coordinate the supply chain under Manufacturer Stackelberg game. This completes the proof.

**Proof of Proposition 12.** In order to achieve coordination, the first goal is to guarantee that the optimal profit of supply chain can reach that in optimal global model ($\pi^{CAS}=\pi^{GO}$). And it’s hard to compare the price of two models directly. We substitute $p^{GO}$ and $t^{GO}$ into formula (18) and (19), and we have: $\pi_{A}^{CAS}\left( p^{GO},t^{GO} \right)=\frac{a(bst_{1}\left( s-h \right)-c_{0}\left( b+\lambda-1 \right)\left( s-h \right)-h\lambda st_{1})\left( b+\lambda-1 \right)^{b+\lambda-1}{(s-h)}^{\lambda-1}}{b^{b}\lambda^{\lambda}s^{b+\lambda}{t_{1}}^{b+\lambda}}-l$ and $\pi_{M}^{CAS}\left( p^{GO},t^{GO} \right)=\frac{a(c_{0}\left( b+\lambda-1 \right)\left( s-h \right)-s\left( b-1 \right)\left( s-h \right)t_{1}+h\lambda st_{1})\left( b+\lambda-1 \right)^{b+\lambda-1}{(s-h)}^{\lambda-1}}{b^{b}\lambda^{\lambda}s^{b+\lambda}{t_{1}}^{b+\lambda}}+l$. To ensure the coordination strategy working, we have to guarantee that $\pi_{A}^{CAS}\left( p^{GO},t^{GO} \right)-\pi_{A}^{AS}=\frac{a\left( bst_{1}\left( s-h \right)-c_{0}\left( b+\lambda-1 \right)\left( s-h \right)-h\lambda st_{1} \right)\left( b+\lambda-1 \right)^{b+\lambda-1}\left( s-h \right)^{\lambda-1}}{b^{b}\lambda^{\lambda}s^{b+\lambda}{t_{1}}^{b+\lambda}}-\frac{as^{\lambda+b-1}{(b-1)}^{b-1}{(1-\lambda)}^{\lambda+b-1}}{b^{b}\lambda^{\lambda}{(\omega s(1-\lambda)+\lambda h(st_{1}-\omega))}^{b-1}{(st_{1}-\omega)}^{\lambda}}-l\geq0$ and $\pi_{M}^{CAS}\left( p^{GO},t^{GO} \right)-\pi_{M}^{AS}=\frac{a\left( c_{0}\left( b+\lambda-1 \right)\left( s-h \right)-s\left( b-1 \right)\left( s-h \right)t_{1}+h\lambda st_{1} \right)\left( b+\lambda-1 \right)^{b+\lambda-1}\left( s-h \right)^{\lambda-1}}{b^{b}\lambda^{\lambda}s^{b+\lambda}{t_{1}}^{b+\lambda}}-\frac{a\left( b-1 \right)^{b}s^{\lambda+b}\left( 1-\lambda\right)^{\lambda+b-1}\left( \left( 1-2\lambda\right)\omega+st_{1}\left( 2\lambda-1 \right) \right)}{b^{b}\lambda^{\lambda}\left( \omega s\left( 1-\lambda\right)+\lambda h\left( st_{1}-\omega\right) \right)^{b}\left( st_{1}-\omega\right)^{\lambda}}+l\geq0$. Thus, we have the range of $l^{1}$: $l^{1}\in\left[ l_{min}^{1}, l_{max}^{1} \right]$ to guarantee that the optimal profit of supply chain can reach that in optimal global model.

$$l_{min}^{1}=\frac{a\left( b-1 \right)^{b}s^{\lambda+b}\left( 1-\lambda\right)^{\lambda+b-1}\left( \left( 1-2\lambda\right)\omega+st_{1}\left( 2\lambda-1 \right) \right)}{b^{b}\lambda^{\lambda}\left( \omega s\left( 1-\lambda\right)+\lambda h\left( st_{1}-\omega\right) \right)^{b}\left( st_{1}-\omega\right)^{\lambda}}-\frac{a\left( c_{0}\left( b+\lambda-1 \right)\left( s-h \right)-s\left( b-1 \right)\left( s-h \right)t_{1}+h\lambda st_{1} \right)\left( b+\lambda-1 \right)^{b+\lambda-1}\left( s-h \right)^{\lambda-1}}{b^{b}\lambda^{\lambda}s^{b+\lambda}{t_{1}}^{b+\lambda}}$$

$$l_{max}^{1}=\frac{a\left( bst_{1}\left( s-h \right)-c_{0}\left( b+\lambda-1 \right)\left( s-h \right)-h\lambda st_{1} \right)\left( b+\lambda-1 \right)^{b+\lambda-1}\left( s-h \right)^{\lambda-1}}{b^{b}\lambda^{\lambda}s^{b+\lambda}{t_{1}}^{b+\lambda}}-\frac{as^{\lambda+b-1}{(b-1)}^{b-1}{(1-\lambda)}^{\lambda+b-1}}{b^{b}\lambda^{\lambda}{(\omega s(1-\lambda)+\lambda h(st_{1}-\omega))}^{b-1}{(st_{1}-\omega)}^{\lambda}}$$

After the coordination of dynamic wholesale price mechanism, it is necessary to ensure that the profit of assemblers and manufacturers is not less than that before negotiation, $\pi_{A}^{CAS}-\pi_{A}^{AS}=\frac{as^{\lambda+b-1}\left( b-1 \right)^{b-1}\left( 1-\lambda\right)^{\lambda+b-1}}{b^{b}\lambda^{\lambda}\left( c_{0}s\left( 1-\lambda\right)+\lambda h\left( st_{1}-c_{0} \right) \right)^{b-1}\left( st_{1}-c_{0} \right)^{\lambda}}-\frac{as^{\lambda+b-1}{(b-1)}^{b-1}{(1-\lambda)}^{\lambda+b-1}}{b^{b}\lambda^{\lambda}{(\omega s(1-\lambda)+\lambda h(st_{1}-\omega))}^{b-1}{(st_{1}-\omega)}^{\lambda}}-l\geq0$ and $\pi_{M}^{CAS}-\pi_{M}^{AS}=\frac{a\left( b-1 \right)^{b}s^{\lambda+b}\left( 1-\lambda\right)^{\lambda+b-1}\left( \left( 1-2\lambda\right)c_{0}+st_{1}\left( 2\lambda-1 \right) \right)}{b^{b}\lambda^{\lambda}\left( c_{0}s\left( 1-\lambda\right)+\lambda h\left( st_{1}-c_{0} \right) \right)^{b}\left( st_{1}-c_{0} \right)^{\lambda}}-\frac{a\left( b-1 \right)^{b}s^{\lambda+b}\left( 1-\lambda\right)^{\lambda+b-1}\left( \left( 1-2\lambda\right)\omega+st_{1}\left( 2\lambda-1 \right) \right)}{b^{b}\lambda^{\lambda}\left( \omega s\left( 1-\lambda\right)+\lambda h\left( st_{1}-\omega\right) \right)^{b}\left( st_{1}-\omega\right)^{\lambda}}+l\geq0$. Then, we can determine the range of $l^{2}$: $l^{2}\in\left[ l_{min}^{2}, l_{max}^{2} \right]$.

$$l_{min}^{2}=\frac{a\left( b-1 \right)^{b}s^{\lambda+b}\left( 1-\lambda\right)^{\lambda+b-1}\left( \left( 1-2\lambda\right)\omega+st_{1}\left( 2\lambda-1 \right) \right)}{b^{b}\lambda^{\lambda}\left( \omega s\left( 1-\lambda\right)+\lambda h\left( st_{1}-\omega\right) \right)^{b}\left( st_{1}-\omega\right)^{\lambda}}-\frac{a\left( b-1 \right)^{b}s^{\lambda+b}\left( 1-\lambda\right)^{\lambda+b-1}\left( \left( 1-2\lambda\right)c_{0}+st_{1}\left( 2\lambda-1 \right) \right)}{b^{b}\lambda^{\lambda}\left( c_{0}s\left( 1-\lambda\right)+\lambda h\left( st_{1}-c_{0} \right) \right)^{b}\left( st_{1}-c_{0} \right)^{\lambda}}$$

$$l_{max}^{2}=\frac{as^{\lambda+b-1}\left( b-1 \right)^{b-1}\left( 1-\lambda\right)^{\lambda+b-1}}{b^{b}\lambda^{\lambda}\left( c_{0}s\left( 1-\lambda\right)+\lambda h\left( st_{1}-c_{0} \right) \right)^{b-1}\left( st_{1}-c_{0} \right)^{\lambda}}-\frac{as^{\lambda+b-1}{(b-1)}^{b-1}{(1-\lambda)}^{\lambda+b-1}}{b^{b}\lambda^{\lambda}{(\omega s(1-\lambda)+\lambda h(st_{1}-\omega))}^{b-1}{(st_{1}-\omega)}^{\lambda}}$$

The range of $l^{1}$ ensure that the profit of supply chain under Assembler Stackelberg game with dynamic wholesale price contract can reach that profit under optimal global model. And the range of $l^{2}$ guarantee that the profit of Assembler and Manufacturer won’t lower than before after coordination. To ensure the success of the coordination strategy, we need to ensure that both conditions are met. And we take the intersection of $l^{1}$ and $l^{2}$. Then we have the range of $l$: $l\in\left[ l_{min}, l_{max} \right]$.

$$l_{min}^{1}=\frac{a\left( b-1 \right)^{b}s^{\lambda+b}\left( 1-\lambda\right)^{\lambda+b-1}\left( \left( 1-2\lambda\right)\omega+st_{1}\left( 2\lambda-1 \right) \right)}{b^{b}\lambda^{\lambda}\left( \omega s\left( 1-\lambda\right)+\lambda h\left( st_{1}-\omega\right) \right)^{b}\left( st_{1}-\omega\right)^{\lambda}}-\frac{a\left( c_{0}\left( b+\lambda-1 \right)\left( s-h \right)-s\left( b-1 \right)\left( s-h \right)t_{1}+h\lambda st_{1} \right)\left( b+\lambda-1 \right)^{b+\lambda-1}\left( s-h \right)^{\lambda-1}}{b^{b}\lambda^{\lambda}s^{b+\lambda}{t_{1}}^{b+\lambda}}$$

$$l_{max}^{1}=\frac{a\left( bst_{1}\left( s-h \right)-c_{0}\left( b+\lambda-1 \right)\left( s-h \right)-h\lambda st_{1} \right)\left( b+\lambda-1 \right)^{b+\lambda-1}\left( s-h \right)^{\lambda-1}}{b^{b}\lambda^{\lambda}s^{b+\lambda}{t_{1}}^{b+\lambda}}-\frac{as^{\lambda+b-1}{(b-1)}^{b-1}{(1-\lambda)}^{\lambda+b-1}}{b^{b}\lambda^{\lambda}{(\omega s(1-\lambda)+\lambda h(st_{1}-\omega))}^{b-1}{(st_{1}-\omega)}^{\lambda}}$$

Thus, the dynamic wholesale price contract can coordinate the supply chain under Assembler Stackelberg game. This completes the proof.
